# Fatigue Induced by Repeated Changes of Direction in Élite Female Football (Soccer) Players: Impact on Lower Limb Biomechanics and Implications for ACL Injury Prevention

**DOI:** 10.3389/fbioe.2021.666841

**Published:** 2021-07-05

**Authors:** Matteo Zago, Sina David, Filippo Bertozzi, Claudia Brunetti, Alice Gatti, Francesca Salaorni, Marco Tarabini, Christel Galvani, Chiarella Sforza, Manuela Galli

**Affiliations:** ^1^Dipartimento di Meccanica, Politecnico di Milano, Milan, Italy; ^2^E4Sport Laboratory, Politecnico di Milano, Lecco, Italy; ^3^Department of Human Movement Sciences, VU University Amsterdam, Amsterdam, Netherlands; ^4^Dipartimento di Scienze Biomediche per la Salute, Politecnico di Milano, Milan, Italy; ^5^IRCCS Fondazione Santa Lucia, Rome, Italy; ^6^Dipartimento di Elettronica, Informazione e Bioingegneria, Politecnico di Milano, Milan, Italy; ^7^Dipartimento di Psicologia, Università Cattolica del Sacro Cuore, Milan, Italy

**Keywords:** women soccer, kinematics, changes of direction, anterior cruciate ligament, injury, fatigue

## Abstract

**Background:**

The etiology of Anterior Cruciate Ligament (ACL) injury in women football results from the interaction of several extrinsic and intrinsic risk factors. Extrinsic factors change dynamically, also due to fatigue. However, existing biomechanical findings concerning the impact of fatigue on the risk of ACL injuries remains inconsistent. We hypothesized that fatigue induced by acute workload in short and intense game periods, might in either of two ways: by pushing lower limbs mechanics toward a pattern close to injury mechanism, or alternatively by inducing opposed protective compensatory adjustments.

**Aim:**

In this study, we aimed at assessing the extent to which fatigue impact on joints kinematics and kinetics while performing repeated changes of direction (CoDs) in the light of the ACL risk factors.

**Methods:**

This was an observational, cross-sectional associative study. Twenty female players (age: 20–31 years, 1st–2nd Italian division) performed a continuous shuttle run test (5-m) involving repeated 180°-CoDs until exhaustion. During the whole test, 3D kinematics and ground reaction forces were used to compute lower limb joints angles and internal moments. Measures of exercise internal load were: peak post-exercise blood lactate concentration, heart rate (HR) and perceived exertion. Continuous linear correlations between kinematics/kinetics waveforms (during the ground contact phase of the pivoting limb) and the number of consecutive CoD were computed during the exercise using a Statistical Parametric Mapping (SPM) approach.

**Results:**

The test lasted 153 ± 72 s, with a rate of 14 ± 2 CoDs/min. Participants reached 95% of maximum HR and a peak lactate concentration of 11.2 ± 2.8 mmol/L. Exercise duration was inversely related to lactate concentration (*r* = −0.517, *p* < 0.01), while neither%HR_*max*_ nor [La^–^]_*b*_ nor RPE were correlated with test duration before exhaustion (*p* > 0.05). Alterations in lower limb kinematics were found in 100%, and in lower limb kinetics in 85% of the players. The most common kinematic pattern was a concurrent progressive reduction in hip and knee flexion angle at initial contact (10 players); 5 of them also showed a significantly more adducted hip. Knee extension moment decreased in 8, knee valgus moment increased in 5 players. A subset of participants showed a drift of pivoting limb kinematics that matches the known ACL injury mechanism; other players displayed less definite or even opposed behaviors.

**Discussion:**

Players exhibited different strategies to cope with repeated CoDs, ranging from protective to potentially dangerous behaviors. While the latter was not a univocal effect, it reinforces the importance of individual biomechanical assessment when coping with fatigue.

## Introduction

Non-contact anterior cruciate ligament (ACL) lesions are the most devastating injuries in female footballers, with an incidence as high as 0.9–2.2 ACL ruptures per 1,000 match hours (2–9 times higher than in males) and accounting for 43% of total time loss due to injuries (Faude et al., 2005; Larruskain et al., 2018), with 32% of female players not returning to competitions (Brophy et al., 2013). Alongside the popularity of women’s football, the rate of ACL injuries continues to rise, despite the advancements in injury mitigation programs (Hewett et al., 2016). This is probably due to an incomplete understanding of the multifactorial nature of ACL injury risk factors (Davidson and Mclean, 2016), suggesting that important factors related to injury are not being incorporated into these programs (Collins et al., 2016).

The etiology of ACL injury in women athletes is multifactorial and results from the interaction of several risk factors (Renstrom et al., 2008; Fox et al., 2014; Nilstad et al., 2014; Barber-Westin and Noyes, 2017; Montalvo et al., 2019), including: narrow intercondylar notch, reduced ACL cross sectional area, unfavorable pelvic-hip-knee-foot alignment, generalized tibio-femoral laxity, greater reliance on stability from ligaments than muscles, low concentric hamstring to quadriceps ratio and hormonal fluctuations. While the multi-joints mechanics of ACL injuries in football games is remarkably similar in male and female players (Della Villa et al., 2020), a prevalence (80% of injuries, on average) of valgus loading and hip internal rotation and abduction was frequently observed (Lucarno et al., 2021), as well as a larger hip and knee flexion (Hewett et al., 2016).

The susceptibility to injury change dynamically (Pol et al., 2019), also due to fatigue. Fatigue is a transient, exercise-induced decline in the pre-match/baseline physiological (and psychological) function that affects both the neurological and musculoskeletal system (Enoka and Duchateau, 2008). When a task involves submaximal contractions, as in many sports activities, the onset of fatigue may not limit the ability to perform the task (Bourne et al., 2019). Yet, fatigue can lead to the generation of inadequate commands in the motor cortex (Taylor et al., 2006; Enoka and Duchateau, 2008), that in turn would impact on joint stability when a perturbation of any component of the system occurs.

The level of evidence supporting the impact of fatigue on the risk factors of ACL injury remains elusive (Barber-Westin and Noyes, 2017; Benjaminse et al., 2019; Bourne et al., 2019). A wide variation was observed in the effects of fatigue on lower limb kinetics, kinematics and muscles activation: for instance, studies have shown that fatigued collegiate football players perform 45-degrees sidestepping maneuvers with greater peak knee and hip flexion angles (Cortes et al., 2013), thus reducing ACL loading; other studies found no significant change in knee flexion or ground reaction forces during cutting after general fatigue protocols (Sanna and O’Connor, 2008; McLean and Samorezov, 2009; Lucci et al., 2011; Cortes et al., 2014), nor changes in hip flexion (Sanna and O’Connor, 2008; Thomas et al., 2011; Zebis et al., 2011; Cortes et al., 2014) or abduction (Cortes et al., 2014). Conversely, other Authors claim that fatigue promotes knee mechanics associated with an augmented risk of ACL injury, that is: increases in peak knee abduction and internal rotation, and/or decrease in maximum knee flexion and hip abduction (Alentorn-Geli et al., 2009; Collins et al., 2016; Mejane et al., 2019); Savage et al. found an increase in knee extension moments in sidestepping actions after a in Australian Football players after a simulated game (Savage et al., 2018). Consistently, in a recent work by our group, the lower limbs kinematics of male non-professional football players was evaluated at the beginning and at the end of 5 min of high-intensity continuous shuttle run at Ciprandi et al. (2018). With respect to the beginning of the test, in the last changes of directions, we observed a 10-degrees drop in peak hip and knee flexion, and a 5-degrees increase of hip external rotation (Zago et al., 2019). This analysis, however, had to be considered limitedly to male non-professional players, lacked dynamic quantities, and did not show what happened throughout the fatiguing process. Agreeing with the aforementioned findings, it was proposed that a progressive accumulation of micro damages due to repeated sub-maximal knee valgus loadings, rather than a single impulsive tensile stress, could be an alternative ACL injury mechanism (Wojtys et al., 2016).

This lack of homogeneity in the existing literature originates from different sources of uncertainty: (i) a minor portion of studies adopting general fatigue models which simulate sport-specific peripheral and central fatigue effects, i.e., current fatigue protocols did not reflect the complexity of physical fatigue that occurs during an actual game (non-ecological validity of testing); (ii) few studies have quantified fatigue in a meaningful way; (iii) few to no data described the effects of the development/onset of fatigue throughout the exercise; (iv) subject groups and athletic tasks analyzed were not homogeneous.

In addition, most of the ACL lesions occur in the first half (Bourne et al., 2019; Della Villa et al., 2020). Therefore, it was hypothesized that it is not the accumulated workload *per se* that affects lower limbs mechanics and in turn the susceptibility to injury, rather the acute workload in relatively short and intense game periods (Pol et al., 2019). Thus, in this study we aimed to investigate whether and how fatigue affects the lower limb biomechanics (kinematics and kinetics waveforms) of uninjured female players while performing repeated changes of direction. The secondary aim is to investigate the relationship of the observed alterations with ACL injury risk factors.

The framework of analysis grounds on repeated turns as those performed in a continuous shuttle run test (Ciprandi et al., 2018; Zago et al., 2018, 2019); 180-degrees changes of direction are sport-specific tasks that can frequently occur during the game (Jones et al., 2016b) imply quick decelerations and high breaking forces to redirect the center of mass momentum (Cortes et al., 2013); such forces can be counteracted by either eccentric muscle contractions and/or passive ligaments loading (Besier et al., 2003; Jones et al., 2017). While jumps and heading actions were seldom the driver of ACL injuries in football (Della Villa et al., 2020; Lucarno et al., 2021), the video analysis of ACL injuries in élite female players revealed that cutting actions like those involved in shuttle run are not only representative of soccer match-play (Greig, 2009), but also a common non-contact injury pattern, especially in defensive “pressing and tackling” situations (Lucarno et al., 2021).

Dependent variables are the time series describing three-dimensional lower limbs kinematics, kinetics and ground reaction forces during the pivoting action. Recent reports demonstrated the feasibility of performing comprehensive analyses of biomechanical parameters over time during turning in female football players by leveraging the Statistical Parametric Mapping (SPM) technique (Hughes-Oliver et al., 2019; Thomas et al., 2021). Modification in dependent variables due to fatigue, and their relationship with the known injury mechanism are going to be addressed as potential markers of an altered injury susceptibility. Based on our previous investigations, we hypothesize that fatigue can significantly alter athletes’ behavior during changes of direction in either of two ways: by pushing lower limbs mechanics toward a pattern close to the non-contact injury mechanism (e.g., reducing knee and hip flexion or increasing knee valgus moment), or alternatively by inducing protective compensatory adjustments.

## Materials and Methods

### Experimental Design

This was an observational, cross-sectional associative study aiming at evaluating the modifications of lower limb mechanics during a physically demanding exercise protocol. It was approved by the Institutional Ethics Committee (protocol n. 06/2020) and met the current ethical standards in Sport and Exercise Science. Participants were recruited on a voluntary basis and took part in the laboratory sessions after signing a written informed consent providing detailed explanation of the risks and benefits of the study.

### Participants

Communication about the research was shared with professional and semi-professional players of the main clubs of the region during the seasons 2018–2019 and 2019–2020. Inclusion criteria were: playing for a women football team competing in the first or second Italian division (“Serie A” or “Serie B”); participating in at least four training sessions per week; no history of previous severe injuries (>28 days of absence from the game) in the 6 months preceding the test; not having sustained any previous ACL or other ligamentous knee injury; being judged by a medical doctor to have no restrictions to sports practice.

**FIGURE 1:**
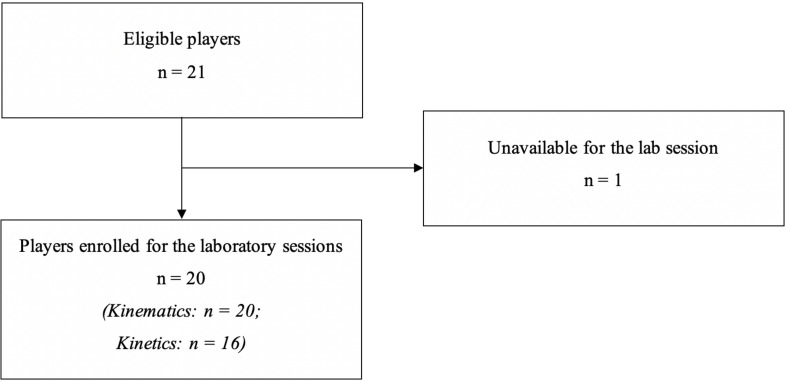
Participants flow and availability.

Twenty players (age range: 20–31 years) were selected and took part in the laboratory tests (Figure 1), that were conducted in two sessions immediately after the end of the 2018–2019 regular season and in the winter break of the following season. The two sessions involved different players, according to their availability. For four players, kinetic data were not available for technical issues. Anthropometrics and physical characteristics are reported in Table 1.

**TABLE 1:** Participants’ anthropometrics and test (continuous shuttle run) performance.

Variable	Unit	Mean	SD	Min	Max
**Anthropometrics**					
Age	years	24.3	3.6	20	31
Height	m	1.65	0.07	1.54	1.75
Body mass	kg	57.5	7.2	47.0	77.0
BMI	kg/m^2^	21.2	1.6	18.4	25.1
**Test performance and physiology**					
Shuttle speed	m/s	2.6	0.2	2.3	2.9
n. CoDs	-	35	16	15	68
Duration	s	153	72	67	305
RPE	-	6.3	1.4	4	9
[La^–^]_*b*_ peak	mmol/L	11.2	2.8	6.7	15.8
HR_*peak*_	bpm	181	6	173	192
HR_*peak*_	%HR_*max*_	95	3	89	99

*BMI, body mass index; [La^–^]_*b*_ peak, peak post-exercise blood lactate concentration; HR, heart rate; n. CoDs, number of changes of direction (sidestepping actions) performed with the dominant limb until exhaustion.*

### Procedures

Laboratory sessions were performed in the morning within a motion capture laboratory equipped with artificial turf, with a controlled ambient temperature of 20–22°C. Participants wore fitting sport clothing and their own indoor soccer shoes. After anthropometrics measurements obtained with a digital stadiometer (standing height to the nearest 1 cm, body mass to the nearest 0.1 kg), the players underwent a 10-min warm-up session led by a licensed strength and conditioning coach involving stretching, jogging, mobilization, lateral hopping, and familiarization with the test.

Immediately following the warm-up routine, athletes started the actual exercise protocol, that was extensively described elsewhere (Ciprandi et al., 2018; Zago et al., 2018, 2019) and involved a continuous 5-m shuttle run test designed to induce continuous 180° turning actions (referred to as changes of direction, CoDs, in the following), repeated with alternated legs to avoid overloading. Running speed was set at an average value of 70% of each participant’s maximum aerobic speed (MAS) and it was dictated by a beat counter. MAS values were previously determined by a YoYo intermittent recovery test (Krustrup et al., 2003). Differently from our previous study on male athletes (Zago et al., 2019), the shuttle run test lasted to exhaustion, until a player missed two beats in a row.

### Data Collection

During the whole test, three-dimensional kinematics was collected at 100 Hz with an 8-cameras SMART-DX optoelectronic system (BTS Bioengineering, Milano, Italy); ground reaction forces data were recorded with two 46.5 × 51.8 cm force platforms (AMTI, Watertown, MA, United States) sampling at 200 Hz. Thirty-seven spherical (diameter: 15 mm) reflective markers were positioned on the following landmarks: radius styloid processes, olecranons, acromia and seventh cervical vertebra; anterior-superior and posterior-superior iliac spines; medial and lateral femoral epicondyles; medial and lateral malleoli; on the shoes in correspondence with the calcaneus and the first and fifth metatarsal heads; four clusters (three-markers each) positioned on the thighs and on the shanks. A 10-s static recording was obtained before the test.

Resting (before exercise) and peak post-exercise blood lactate concentration ([La^–^]_*b*_) was determined with a portable lactate analyzer (Lactate Pro 2, Cosmed, Roma, Italy) every 2 min after the test, until a decrease in the measure was observed. Heart rate (HR) during the exercise was also recorded (Polar Electro, Espoo, Finland). After the shuttle test, subjects provided a rating of their perceived exertion (RPE) on the 0–10 Borg scale.

### Data Analysis

Inverse kinematics and dynamics computations were undertaken in Visual 3D (v. 6.01.36, C-Motion, Germantown, MD, United States), using a 24° of freedom pelvis and lower extremities cluster-based model (Cappozzo et al., 1997); inverse kinematics was computed after constraining joints’ translational degrees of freedom. Raw markers trajectories and forces were low-pass filtered with a fourth-order Butterworth filter with a cut-off frequency of 18 Hz (Smeets et al., 2020). We obtained the time course of a set of 23 variables during the ground contact phase:

• pelvis rotation and lower limbs three-dimensional joint angles (pivoting limb hip, knee and ankle),
• centre of mass (CoM) velocity in the running direction (Mapelli et al., 2014),
• three-dimensional ground reaction forces,
• three-dimensional internal moments of force (pivoting limb hip, knee, ankle) through inverse dynamics

Joint moments and GRF were both normalized to body mass. After excluding the first three CoDs (used by the athletes to adjust themselves to the running pace), kinematics and kinetics waveforms during the ground contact phase of the dominant side (i.e., limb preferentially used to kick the ball) pivoting limb were resampled to 101 time points.

### Statistical Analysis

**FIGURE 2:**
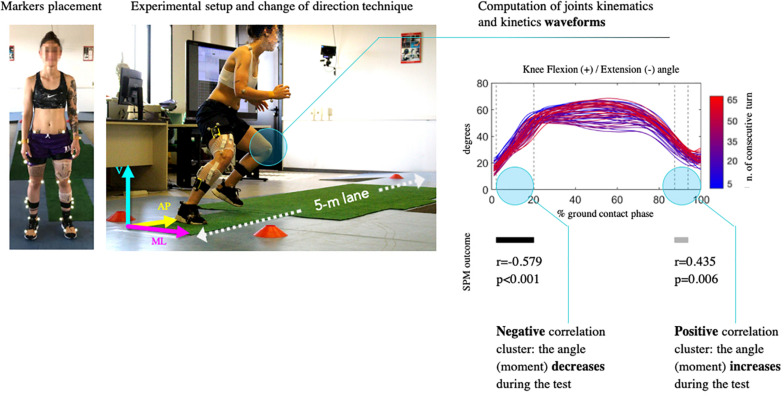
**(Left)** Experimental setup. Kinematic and kinetic curves were evaluated during the ground support phase of all the changes of directions performed throughout the test with the dominant limb; direction of ground reaction forces was displayed (ML, mediolateral; AP, anteroposterior; V, vertical). **(Right)** Statistical analysis procedure. A linear regression for continuous variables was then applied between the time series of the angle and the number of consecutive turn (in the example plot, the curves fade from blue to red as the number of changes of direction increases). Statistical parametric mapping (SPM) returned clusters of significantly positive (gray bars) or negative (black bars) correlation.

Statistical analyses were performed within Matlab (v. 2019b, Mathworks Inc., Natwick, WY, United States). Variations in dependent variables (i.e., kinematics and kinetics waveforms) throughout the test were tested using a SPM^1^ approach. SPM analysis enabled exploration of the relationships between continuous parameters and a discrete value without having to impose the temporal focus bias (Pataky et al., 2013). This method was used to compute the linear correlation between the number of consecutive turns and the time-series of the 23 kinematic and kinetic variables. As described in Figure 2, we considered only significant (α = 0.01) scalar output clusters (i.e., adjacent periods of significant correlation) in the first 20% of the ground support phase. This was done as non-contact ligamentous injuries usually occur within the first 80 ms after initial contact, namely during the landing and weight acceptance phase (Koga et al., 2018; Della Villa et al., 2020). This analysis was repeated separately for each participant and variable. Pearson’s linear correlation coefficients (r) between physiological and exercise-related discrete parameters were also calculated.

## Results

### Exercise Intensity

A total of 699 CoDs were analyzed: participants completed between 15 and 68 CoDs with the preferred limb before exhaustion (and an equal amount with the non-preferred limb), corresponding to 14 (SD: 2) CoDs per minute. Average ground contact time was 0.4 (0.2) s. The shuttle test was performed at an average speed of 2.6 (0.2) m/s and lasted on average 153 (72) s; reaching 95% of the maximum HR (%HR_*max*_) and with a peak post-exercise lactate concentration of 11.2 (2.8) mmol/L. Reported exertion substantially varied among individuals (4–9).

Exercise duration was inversely related to lactate concentration (*r* = −0.517, *p* < 0.01), that is: the lower the running speed, the higher the lactate concentration (and the longer the exercise duration, *r* = −0.579, *p* < 0.01). Neither%HR_*max*_ nor [La^–^]_*b*_ nor RPE were correlated with test duration before exhaustion (*p* > 0.05).

### Kinematics Adaptations Due to Repeated CoDs

**FIGURE 3:**
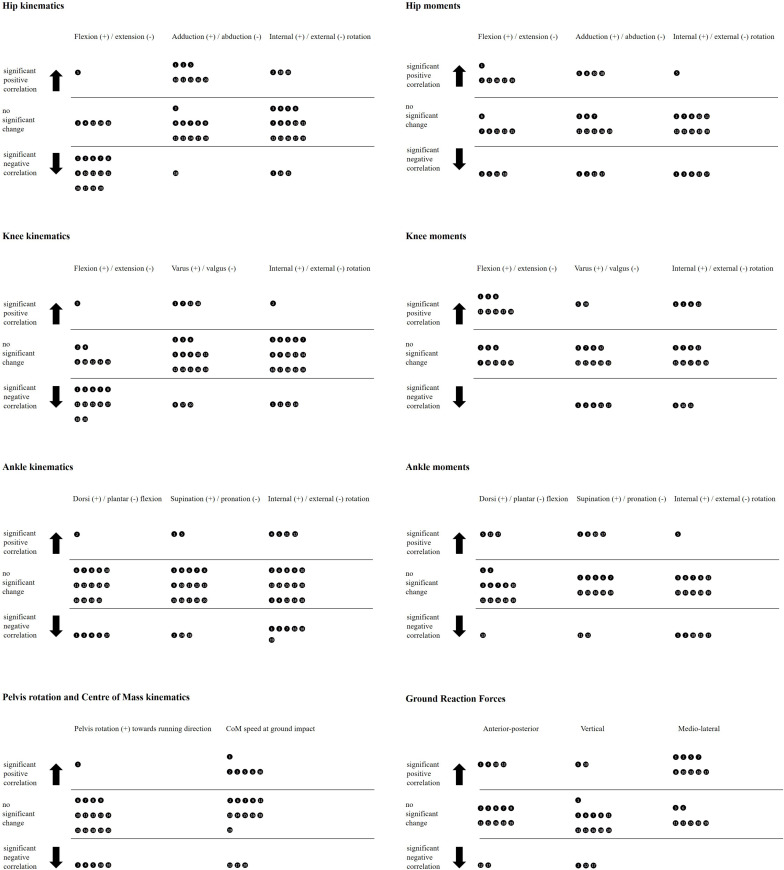
Results overview. Each circle represents a participant and accounts for the correlation between the number of consecutive turns and the kinematics **(Left)** and kinetics **(Right)** of the pivoting limb during the ground contact phase. Positive (negative) correlation means that the angle increased (decreased) throughout the exercise, toward the direction indicated by the “±” symbols; for example, a negative correlation in hip flexion angle indicates a progressive reduction of the hip flexion angle during the test.

All the players showed statistically significant modifications of lower limbs kinematics in at least two of the considered variables. Figure 3 provides a visual summary of the results: the most evident trend in joints kinematics was a reduction in hip and knee flexion angles in 14 (70%, *r* ranging from −0.752 to −0.844, *p* < 0.001) and 12 (60%, *r* = −0.520 to −0.928, *p* < 0.01) of the participants, respectively. These two angles increased during the test in only 1/20 players (Player 5). More specifically, the observed drop in hip flexion at initial contact varied from about 10° (Players 6 and 13) up to 25° (Player 20). The reduction in knee flexion at initial contact ranged from about 5° (Players 6 and 18) to over 20° (Player 11).

Hip abduction decreased in 8 players (40%, *r* = 0.617 to 0.912, *p* < 0.01) of 5–10° at initial contact (Players 1, 2, 5, 15, and 20) and did not change in 11 players. On the frontal and transverse planes, knee and ankle angles were primarily unchanged during the test, and a clear trend could not be identified. In particular, knee adduction and rotation angles did not change in 70 and 75% of the players, respectively. Pelvis rotation toward the new running direction was reduced in 5/20 players (*r* = −0.398 to −0.920, *p* < 0.01) and unchanged in 14/20, while CoM speed at ground contact increased in 6 players from 0.7–0.8 to 1.2–2.1 m/s (Players 1, 2, 3, 5, 8, and 10, *r* = 0.751 to 0.801, *p* < 0.001) and was unchanged in 11.

Participants’ full kinematics and kinetics waveforms during the ground support phase of all the performed CoDs are available as Supplementary material, as well as the corresponding SPM output; Supplementary Tables 1, 2 report the directional changes for each player and variable.

### Kinetics Adaptations Due to Repeated CoDs

Four players showed significant concurrent changes in 75% or more variables (only 1/16 players with valid kinetic data displayed no significant changes). However, a distinct trend was not noticeable in most cases (Figure 3). Two exceptions were: mediolateral ground reaction force increased in 9 players (*r* = 0.554 to 0.912, *p* = <0.01; e.g., it increased from about 0 Nm/kg at 10% of the ground support phase to nearly 0.1 Nm/kg in Players 13 and 15) and was unchanged in 7; knee extension moment decreased in 8 (50%, *r* = −0.574–0.873) and was unchanged in the other players. Knee valgus moment increased in 5 and reduced in 2 players, with changes between early and late turns ranging from −2.5 and 0.2 Nm/kg at about 10% of the ground contact phase.

Hip extension moment also decreased in 6/16 players by around 1.5 Nm/kg at 10% of the ground contact phase (*r* = −0.703 to 0.801, unchanged in 6). Hip and ankle internal rotation moments decreased in 5/16 players. The other ankle moments were unchanged in 75% or more players.

## Discussion

The repetition of turning actions (180°-CoDs) induced alterations in lower limb kinematics in all the tested players, and in lower limb kinetics in 85% of the players. The nature and extent to which lower limbs mechanics was altered throughout the test were highly variable among individuals. Biomechanical modifications mostly occurred in the sagittal plane, but adaptations in the coronal and transverse planes were also detected. A subset of participants showed a drift of pivoting limb kinematics that tightly matches with the known ACL injury mechanism; other players displayed less definite, minimal or even opposed behaviors.

The current study introduced a focal change in the analysis of fatigue effects: moving away from the traditional “pre- vs. post-fatigue” paradigm, we assessed the biomechanical waveforms throughout the whole exercise, by exploiting the capabilities of SPM. This approach tackles one of the uncertainty sources of existing studies (no description of fatigue effects during exercise, see Chapter 1) and allowed to detect deteriorations in biomechanics due to sub-maximal repetitive knee loadings replicated over a short time span while performing changes of directions (Wojtys et al., 2016), or to investigate between-limb differences in 180-degrees turns in female players (Thomas et al., 2021). Notably, the adopted methodology does not rely on arbitrarily defined discrete parameters, rather it consistently analyses the whole time course of a large set of kinetic and kinematic time series (Hughes-Oliver et al., 2019).

When discussing our findings, we will embrace an injury perspective recently proposed by Pol et al. (2019): an athlete’s organism constantly adapts to emerging constraints at different timescales (weeks to minutes) to maintain stability in presence of perturbations. Workload (in our case, CoDs repetition) is a type of constraint that influences the susceptibility to injury (Pol et al., 2019). As the workload is changing over time, also the susceptibility to injury changes dynamically over short timescales (Meeuwisse et al., 2007) (e.g., during the shuttle test). This happens because fatigue might impair the muscle function and motor coordination (Wojtys et al., 2016; Pol et al., 2019), producing a change of the biomechanical variables. What is of interest here is to discern whether such fatigue-induced adaptations could place tested female players at a higher risk of tearing their ACL during cutting actions.

### Changes in Lower Limbs Biomechanics

The most represented kinematic pattern we found was a progressive reduction in hip and knee flexion angle at initial contact, observed concurrently in 10/20 players. In 5 of these 10, a reduction of hip abduction (a more adducted hip) was also observed. Three players showed a trend toward knee valgus, and six players showed a progressively externally rotated foot. In six players the approaching CoM speed increased toward the end of the test (at constant shuttle speed). Only three players did not show a reduction of either the knee or the hip flexion angle.

The relevance of these results to injury risk evaluation is two-fold: first, kinematic changes confirm the trend observed in our previous study on male athletes (Zago et al., 2019) and in other investigations on fatigue-induced alterations in jumps and landing tasks (McLean et al., 2004; Mejane et al., 2019; Smeets et al., 2020). Second, for a fraction of the participants, this scenario depicts a fatigue-induced alteration in body segments arrangement that drives in the direction of the well-known ACL injury mechanism (Renstrom et al., 2008; Fox et al., 2014; Hilibrand et al., 2015; Hewett et al., 2016; Benjaminse et al., 2019; Della Villa et al., 2020): reduced hip and knee extension, hip less abducted and internally rotated, knee valgus and internal (or external) rotation, flat foot strike [plantarflexed and pronated (David et al., 2017)] and external foot rotation; external knee moments of abduction, flexion (causing knee compartment compression) and rotation.

In particular, an extended knee (0°–40°) is often referred to as a potentially deleterious posture, as it could increases the shear force on the ACL (Siegel et al., 2012; Cortes et al., 2013). It could be that, in this position, hamstrings struggle in counteracting quadriceps’ anterior tibial shear force due to a wider hamstring insertion (elevation) angle. In addition, the higher CoM approach speed influences body momentum and thus the loads absorbed during the CoD. As the average shuttle speed had to be maintained constant, we hypothesize this might be due to a reduced braking contribution in the penultimate step (Jones et al., 2016a; David et al., 2018; Dos’Santos et al., 2020; Staynor et al., 2020). A plausible motivation of lower knee and hip flexion could be that fatigued muscles ([La^–^]_*b*_ > 11 mmol/L) might have become progressively less effective in dissipating impact forces through eccentric action, thus transmitting load to passive structures (Alentorn-Geli et al., 2009; Cortes et al., 2013; Weiss and Whatman, 2015; Zago et al., 2019).

A concurrent macroscopic result of our study was that a univocal trend in kinematics and kinetic changes could not be found. In 11/14 kinematic variables we did not detect a significant correlation at initial contact. There were a fraction of players showing minimal changes or even protective adaptations, suggesting that regulatory attempts to prevent harms can arise in response to a perturbed (fatigued) state. For instance, a reduction of knee extension moment was found in 8/16 players. This might be due to fatigued knee extensors muscles that forced to adopt a different strategy to negotiate the CoD. In this sense, our findings did not show a mechanistic link between fatigued muscles and an increase in the moments around the knee joints, as hypothesized by Savage et al. for 45-degrees sidestepping and crossover cutting tasks (Savage et al., 2018). On the contrary, we have observed a protective regulation contrasting a potential increase of knee external flexion moment, that in turn was indicated as a contributor to ACL injury (Savage et al., 2018).

In brief, these findings suggest that some players could compensate the impairment induced by acute workload (increase of physiological demands) with a sort of “safety strategy” to preserve the integrity of passive structures and safely dissipate energy during CoDs. The reduction of knee adduction moment for 5/16 players, the shift toward a more adducted knee observed in four players and the increase in hip and knee flexion angles observed in Player 5 seem to support this view.

### Fatigue Protocol Intensity and Inter-Individual Variability

Extensive measures of internal load (95% HR_*max*_, [La^–^]_*b*_ far above the aerobic threshold, RPE) and external load (∼2.6-min bouts, ∼14 CoDs/min) denoted an high-intensity exercise, with a substantial muscle load due to repeated decelerations and accelerations (Ciprandi et al., 2018; Zago et al., 2019). While the accumulation of muscle lactate during intense exercise do not *per se* cause fatigue during exercise, it appears that they contribute to fatigue development (Tesch et al., 1978; Bangsbo and Hostrup, 2019). With respect to existing associative studies adopting general fatigue protocols (Mejane et al., 2019; Smeets et al., 2020), a fatigued state was reached through the repetition of sport-specific actions, thus comparatively increasing the ecological validity of testing (Enoka and Duchateau, 2008). That said, continuous shuttle is not a game running pattern, and with respect to a whole game, the frequency of CoDs performed was higher than the ∼8 (SD: 3) CoDs/min reported by Bloomfield et al. (2007). However, in laboratory conditions this shuttle test allowed internal load parameters to match the activity profile of team sports (Ciprandi et al., 2018), and in particular of high-intensity short (<5 min) periods in small-sided games and demanding intense match-play phases (Randers et al., 2010; López-Fernández et al., 2018; Riboli et al., 2020).

Even with individualized exercise load (70% of players’ MAS), each player showed a distinct pattern of alterations. It is unlikely that the underlying reason would just lie in the differences of the number of performed CoDs. For instance, Players 1, 5, and 17 completed 67–68 shuttles till exhaustion and showed several (but diverse) alterations in kinematics and kinetics. However, there were also cases like Player 2 who performed as little as half shuttles and still showed large modifications during the test. At the same time, players who performed similar amounts of CoDs (Player 10 and 11) at different speed (2.9 vs. 2.4 m/s) showed similar biomechanical patterns when coping with the exercise, even though perceived (RPE = 7) and measured (%HR_*max*_ = 93–94%, [La^–^]_*b*_ = 9.3–11.9 mmol/L) internal load was substantially the same.

Of note, there is currently a marked gap in terms of resources, staff and equipment between a top first-division Italian club and a mid-ranked second-division team. This inevitably impacted upon differences between athletes in physical fitness and familiarity with injury mitigation programs. However, the big picture emerging from this variety of baseline conditions suggests that “fatigue alter the injury risk of athletes in different ways and proportions” as recently stated by Verschueren et al. (2020), and that biomechanical adaptations to fatigue do not depend only on workload, but also on a plethora of intrinsic and extrinsic factors as competition level, fitness status, type and frequency of previous neuromuscular preventive training that together compose a unique set of physiological (and psychological) features (Verschueren et al., 2020).

### Fatigue, Injury Risk, and Practical Implications

Recent meta analyses concluded that there is no clear association between fatigue (workload in training and competition) and ACL injury (Benjaminse et al., 2019; Bourne et al., 2019). Globally, our results seem to support this view. However, our initial hypothesis by which fatigue induced by repeated CoDs over a short timescale (minutes) can potentially generate hazardous alterations in lower limbs biomechanics was supported in a subset of the assessed cohort. For those players, the identified changes at initial contact were reported to increase the risk of ACL injury (Weiss and Whatman, 2015; Staynor et al., 2020), dangerously bringing an athlete closer to the so called “position of no return” (Hilibrand et al., 2015). While this was not a univocal effect, we believe that it cannot be ignored: testing in fatigued conditions should be considered for screening purposes and to properly evaluate an athlete’s neuromuscular control (Smeets et al., 2020; Verschueren et al., 2020). In addition, targeted neuromuscular exercises (i.e., single-leg landing and/or cutting, proprioception drills) should be performed in a perturbed (fatigued) conditions, whilst in a controlled environment. This would add ecological validity to the intervention, and we argue that would elicit adaptations in a context more closely matching the hazard of critical game actions.

We would like to point out that this is an associative study: we found a link between acute fatigue development during exercise and, in some cases, behaviors that can be associated to ACL injuries. This does not directly imply that fatigue alone is responsible of an injury (Alentorn-Geli et al., 2009), but that when such behaviors are detected, they should be tackled in a preventive perspective. In fact, in the current and in all the previous laboratory studies, fatigue was never enough to induce a non-contact ACL injury: an inciting event must occur to trigger the causal pathway leading to ligamentous rupture (Hewett et al., 2016). This could originate from external factors as an opponent’s feint or a cognitive perturbations, combined with weather and playing surface conditions (Rafeeuddin et al., 2016; Bourne et al., 2019; Monfort et al., 2019; Della Villa et al., 2020).

### Limitations

As the mechanical and neuromuscular demands of changes of directions are angle-dependent (Dos’Santos et al., 2017), results on 180°-CoDs does not immediately generalize to other CoD angles. In addition, it is possible that in real game conditions the same motor actions could be performed differently, due to a higher cognitive load (distractions, decision making process, etc.). In the current study, laboratory conditions were used to model a simplified environment in which the specific effect of fatigue could be investigated. In addition, we did not address muscles activation, which could have shed light on the alterations (timings, intensity) of muscle groups leading to the observed changes.

Participants were wearing their own pairs of indoor soccer shoes; while this was a subject-wise analysis, we are aware that different shoe-surface mechanical coupling due to specific shoe models might in principle impact on the adaptations to exercise and constitute an additional source of inter-individual variability.

Only the final plant step of the change of directions has been analyzed; it would be interesting to address in future work whether fatigue influences the role of the penultimate foot contact within a change of direction (Jones et al., 2016a).

This study used multiple linear regression for continuous time series to assess the change of kinematics and kinetics waveforms during exercise (Pataky et al., 2013). However, fatigue-mediated biomechanical modifications could manifest themselves through non-linear dynamics. We encourage further research focusing on how this phenomenon could be mathematically handled.

Lastly, while being a strictly defined sample, the players recruited belonged to different teams. We cannot exclude that a more homogeneous (and possibly larger) cohort would have returned more consistent results due to even exposure to injury prevention and athletic training programs.

## Conclusion

In female footballers, fatigue induced by repeated turns altered lower limbs mechanics. The fatigue effects were evaluated by assessing the biomechanical waveforms throughout the exercise, overcoming the conventional “pre- vs. post-” design. Biomechanical changes mainly occurred in the sagittal plane, with only a subset of participants also showing modifications in the coronal and transverse planes. Accordingly, the tested players demonstrated different strategies to cope with the exercise, ranging from protective to potentially dangerous behaviors (i.e., resembling the ACL injury mechanism). While not being in itself an isolated risk factors, we concluded that in some of the tested players fatigue affected the musculoskeletal system in a way that may contribute to bring an athlete toward an unsafe condition.

As the physiological, and in turn neuromuscular and biomechanical adaptations to fatigue were strongly subject-specific (Verschueren et al., 2020), these findings reinforce the importance of individual biomechanical assessment when coping with perturbations, as it is fatigue. Biomechanical and neuromuscular risk factors can be modified (Hewett et al., 2016): to ensure the highest effectiveness, injury mitigation programs should be tailored to the individual responses to sport-specific constraints, such as fatigue.

## Data Availability Statement

The original contributions presented in the study are included in the article/Supplementary Material, further inquiries can be directed to the corresponding author.

## Ethics Statement

The studies involving human participants were reviewed and approved by Comitato Etico Politecnico di Milano n. 6/2020. The patients/participants provided their written informed consent to participate in this study.

## Author Contributions

MZ: study conception and design, data collection and statistical analysis, and initial draft of the manuscript. CB, AG, and FS: experimental setup and laboratory sessions, data collection and preparation, and data analysis. SD and FB: data collection, discussion on results, and critical revision and writing of selected parts of the manuscript. MG and CS: supervision, data interpretation, and critical revision of the study methods and results. MT and CG: supervision and critical revision of the manuscript. All the authors approved the final version of the manuscript.

## Conflict of Interest

The authors declare that the research was conducted in the absence of any commercial or financial relationships that could be construed as a potential conflict of interest.

## Footnotes

1 www.spm1d.org
