# Supplementary material for: Fatigue Induced by Repeated Changes of Direction in Élite Female Football (Soccer) Players: Impact on Lower Limb Biomechanics and Implications for ACL Injury Prevention
Source: Front Bioeng Biotechnol. 2021 Jul 5;9:666841. doi: 10.3389/fbioe.2021.666841 (PMC8287513; doi:10.3389/fbioe.2021.666841)
Supplement: Supplementary file 1 [file Data_Sheet_1.PDF]

Supplementary Material

1 Supplementary Tables

Table 1_Supplementary: summary kinematics outcomes for each participant. The “+/-” symbol indicates a positive/negative correlation of the selected variable (corresponding waveform during the ground contact phase) with the number of consecutive turns performed.

**Player**	**Shuttle speed (m/s)**	**$\%HR_{max}$**	**$[La^-]_b$**	**RPE**	**n**	**Pelvis**	**Hip**			**Knee**			**Ankle**			**CoM speed**
						**R**	**F**	**A**	**R**	**F**	**V**	**R**	**F**	**S**	**R**	
P1	2.53	91.8	6.8	6	68	+	-	+	-	-	+	-	-	+	-	+
P2	2.68	90.9	11.1	7	23	nc	-	+	+	nc	nc	+	+	-	nc	+
P3	2.57	92.9	11.8	6	27	-	nc	nc	nc	-	nc	nc	-	nc	-	+
P4	2.66	98.0	14.4	7	39	-	nc	nc	nc	nc	nc	nc	-	nc	+	nc
P5	2.33	95.2	6.7	7	67	-	+	+	nc	+	nc	nc	-	+	+	+
P6	2.92	92.9	9.6	8	15	nc	-	nc	nc	-	nc	nc	nc	nc	nc	nc
P7	2.58	98.4	10.3	6	27	nc	-	nc	nc	-	+	nc	nc	nc	-	nc
P8	2.58	96.4	11.2	8	39	nc	-	nc	nc	nc	nc	nc	nc	nc	nc	+
P9	2.58	89.2	15.3	9	28	nc	-	nc	nc	-	-	nc	nc	nc	nc	nc
P10	2.92	92.8	9.3	7	35	-	-	+	nc	nc	nc	nc	nc	nc	nc	+
P11	2.40	94.3	11.9	7	36	nc	-	+	nc	-	nc	-	nc	nc	+	nc
P12	2.53	92.4	14.9	3	23	nc	nc	nc	nc	nc	nc	-	nc	nc	+	-
P13	2.48	91.1	11.2	5	26	nc	-	nc	nc	-	+	nc	nc	nc	nc	nc
P14	2.53	99.3	6.9	5	30	nc	nc	nc	-	nc	nc	nc	nc	-	nc	nc
P15	2.53	99.8	14.7	7	40	nc	-	+	-	-	nc	-	nc	nc	nc	nc
P16	2.33	94.2		4	50	nc	-	+	nc	-	nc	nc	nc	nc	-	nc
P17	2.33	na	9.3	6	67	nc	-	nc	nc	-	-	nc	-	nc	nc	-
P18	2.57	97.6	15.8	6	18	-	nc	-	+	-	+	nc	nc	-	-	nc
P19	2.68	97.8	9.9	7	18	nc	-	nc	nc	nc	nc	nc	nc	nc	-	nc
P20	2.57	97.3	11.4	5	23	nc	-	+	+	-	-	nc	nc	nc	nc	-
Summary					+	1	1	8	3	1	4	1	1	2	4	6
					nc	14	5	11	14	7	13	15	14	15	10	11
					-	5	14	1	3	12	3	4	5	3	6	3

CoM: centre of mass; nc: no significant correlation; n: number of changes of direction performed with the dominant limb before exhaustion. A: + towards adduction; F: + towards flexion (dorsiflexion for the ankle joint); R: rotation (+ towards the running direction for the pelvis, internal for the other joints); S: + towards supination; V: + towards varus (adducted) knee.

Table 2_Supplementary: summary kinetics outcomes for each participant. The “+/-” symbol indicates a positive/negative correlation of the selected variable (corresponding waveform during the ground contact phase) with the number of consecutive turns performed.

**Player**	**Shuttle speed (m/s)**	**$\%HR_{max}$**	**$[La^-]_b$**	**RPE**	**n**	**Hip**			**Knee**			**Ankle**			**GRF**		
						**F**	**A**	**R**	**F**	**V**	**R**	**F**	**S**	**R**	**Ap**	**Vr**	**Ml**
P1	2.53	91.8	6.8	6	68	+	-	-	+	-	+	nc	+	-	+	nc	+
P2	2.68	90.9	11.1	7	23	+	-	nc	nc	-	+	nc	nc	-	nc	-	+
P3	2.57	92.9	11.8	6	27	-	nc	-	+	nc	nc	nc	nc	nc	nc	nc	nc
P4	2.66	98.0	14.4	7	39	na											
P5	2.33	95.2	6.7	7	67	-	+	+	nc	+	-	+	nc	+	+	+	+
P6	2.92	92.9	9.6	8	15	nc	nc	-	nc	-	+	nc	nc	nc	nc	nc	nc
P7	2.58	98.4	10.3	6	27	nc	nc	nc	nc	nc	nc	nc	nc	nc	nc	nc	+
P8	2.58	96.4	11.2	8	39	nc	+	nc	+	nc	nc	nc	+	nc	nc	nc	+
P9	2.58	89.2	15.3	9	28	na											
P10	2.92	92.8	9.3	7	35	-	+	nc	nc	+	-	nc	+	-	+	+	+
P11	2.40	94.3	11.9	7	36	nc	nc	nc	+	-	-	nc	-	-	nc	nc	nc
P12	2.53	92.4	14.9	3	23	+	nc	nc	+	nc	nc	+	-	nc	-	-	nc
P13	2.48	91.1	11.2	5	26	nc	-	-	nc	nc	+	-	nc	nc	+	nc	+
P14	2.53	99.3	6.9	5	30	na											
P15	2.53	99.8	14.7	7	40	nc	nc	nc	nc	nc	nc	nc	nc	nc	nc	nc	nc
P16	2.33	94.2	na	4	50	+	nc	nc	+	nc	nc	nc	nc	nc	nc	nc	+
P17	2.33	na	9.3	6	67	+	-	-	+	-	nc	+	+	-	-	-	+
P18	2.57	97.6	15.8	6	18	-	+	nc	+	nc	nc	nc	nc	nc	nc	nc	nc
P19	2.68	97.8	9.9	7	18	+	nc	nc	nc	nc	nc	nc	nc	nc	nc	nc	nc
P20	2.57	97.3	11.4	5	23	na											
Summary					+	6	4	1	8	2	4	3	4	1	4	2	9
					nc	6	8	10	8	9	9	12	10	10	10	11	7
					-	4	4	5	0	5	3	1	2	5	2	3	0

Ap: antero-posterior (running) direction; Ml: medio-lateral direction; na: not available; nc: no significant correlation; n: number of changes of direction performed with the dominant limb before exhaustion; Vr: vertical direction. A: + towards adduction; F: + towards flexion (dorsiflexion for the ankle joint); R: rotation (+ towards running direction for the pelvis, internal for the other joints); S: + towards supination; V: + varus moment.

2 Supplementary Figures

In the following, the complete set of joint kinematics (left) and kinetics (right) curves are displayed in separate pages for each participant. Waveforms color shifts from blue to red based on the corresponding number of the change of direction performed (blue: exercise start, red: exhaustion).

The bars behind the plot represent time windows of positive (gray) or negative (black) correlation of the curves values with the number of repetitions, evaluated through Statistical Parametric Mapping. The value and significance of correlation are also displayed.

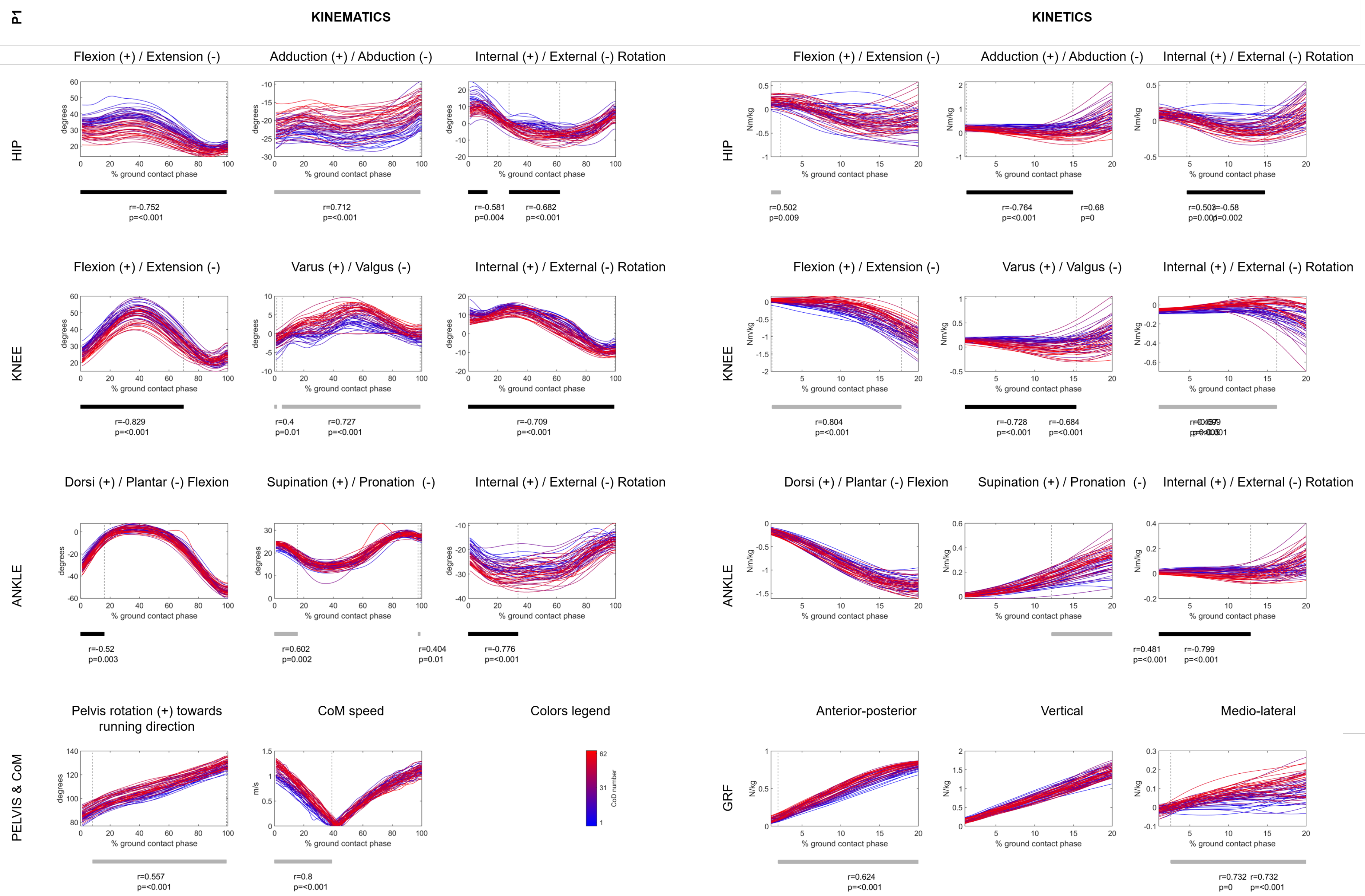
P1
KINEMATICS
KINETICS
HIP
KNEE
ANKLE
PELVIS & CoM
GRF
Flexion (+) / Extension (-)
Adduction (+) / Abduction (-)
Internal (+) / External (-) Rotation
Varus (+) / Valgus (-)
Dorsi (+) / Plantar (-) Flexion
Supination (+) / Pronation (-)
Pelvis rotation (+) towards running direction
CoM speed
Colors legend
Anterior-posterior
Vertical
Medio-lateral
degrees
Nm/kg
m/s
N/kg
% ground contact phase
CoD number
r=-0.752 p=<0.001
r=0.712 p=<0.001
r=-0.581 p=0.004
r=-0.682 p=<0.001
r=0.502 p=0.009
r=-0.764 p=<0.001
r=0.68 p=0
r=-0.829 p=<0.001
r=0.4 p=0.01
r=0.727 p=<0.001
r=-0.709 p=<0.001
r=0.804 p=<0.001
r=-0.728 p=<0.001
r=-0.684 p=<0.001
r=-0.52 p=0.003
r=0.602 p=0.002
r=0.404 p=0.01
r=-0.776 p=<0.001
r=0.481 p=<0.001
r=-0.799 p=<0.001
r=0.557 p=<0.001
r=0.8 p=<0.001
r=0.624 p=<0.001
r=0.732 p=0
r=0.732 p=<0.001

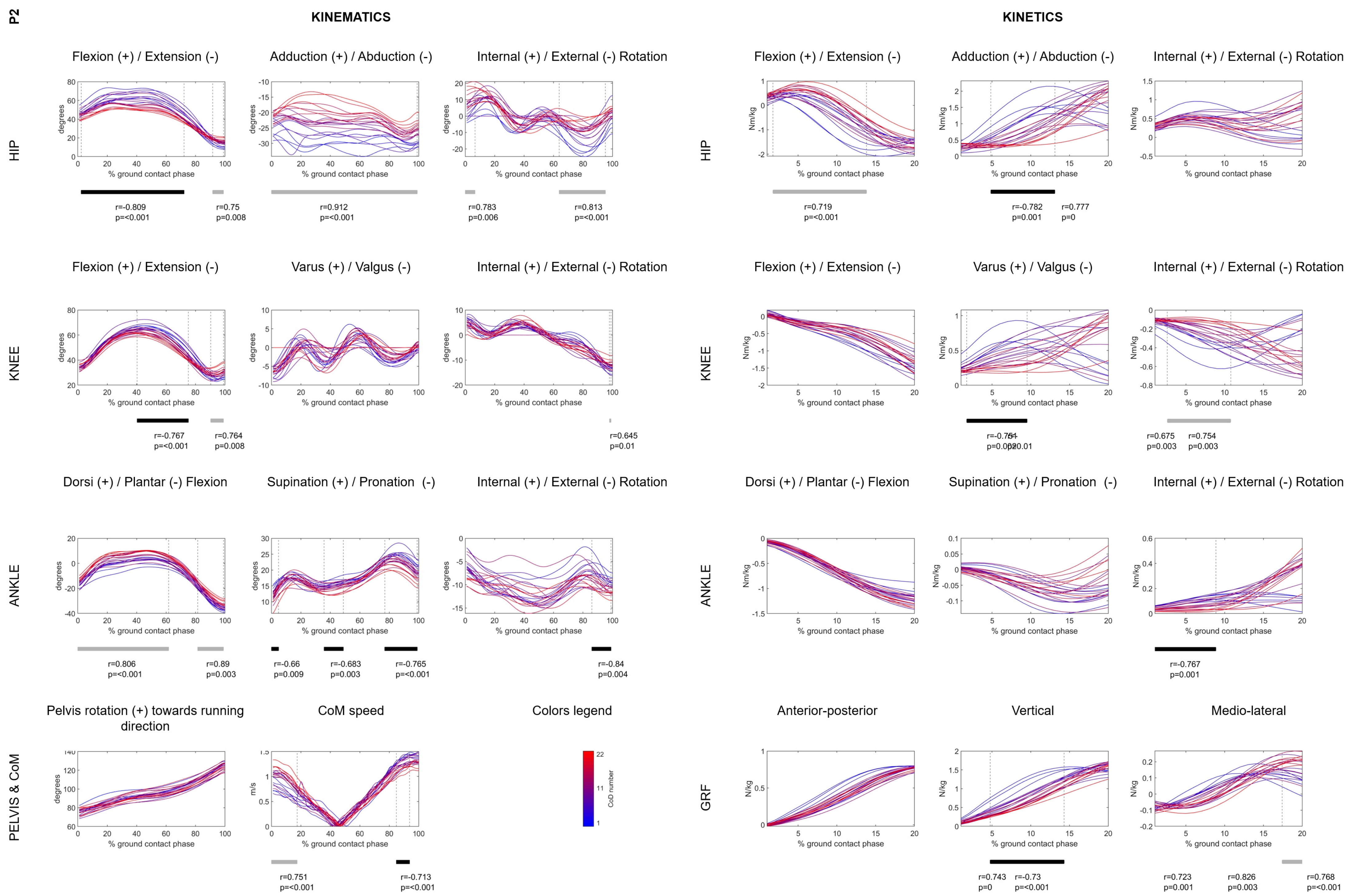
P2
KINEMATICS
KINETICS
HIP
KNEE
ANKLE
PELVIS & CoM
GRF
Flexion (+) / Extension (-)
Adduction (+) / Abduction (-)
Internal (+) / External (-) Rotation
Varus (+) / Valgus (-)
Dorsi (+) / Plantar (-) Flexion
Supination (+) / Pronation (-)
Pelvis rotation (+) towards running direction
CoM speed
Colors legend
CoD number
Anterior-posterior
Vertical
Medio-lateral
degrees
Nm/kg
N/kg
m/s
% ground contact phase
r=-0.809 p=<0.001
r=0.75 p=0.008
r=0.912 p=<0.001
r=0.783 p=0.006
r=0.813 p=<0.001
r=0.719 p=<0.001
r=-0.782 p=0.001
r=0.777 p=0
r=-0.767 p=<0.001
r=0.764 p=0.008
r=0.645 p=0.01
r=0.675 p=0.003
r=0.754 p=0.003
r=0.806 p=<0.001
r=0.89 p=0.003
r=-0.66 p=0.009
r=-0.683 p=0.003
r=-0.765 p=<0.001
r=-0.84 p=0.004
r=-0.767 p=0.001
r=0.751 p=<0.001
r=-0.713 p=<0.001
r=0.743 p=0
r=-0.73 p=<0.001
r=0.723 p=0.001
r=0.826 p=0.003
r=0.768 p=<0.001

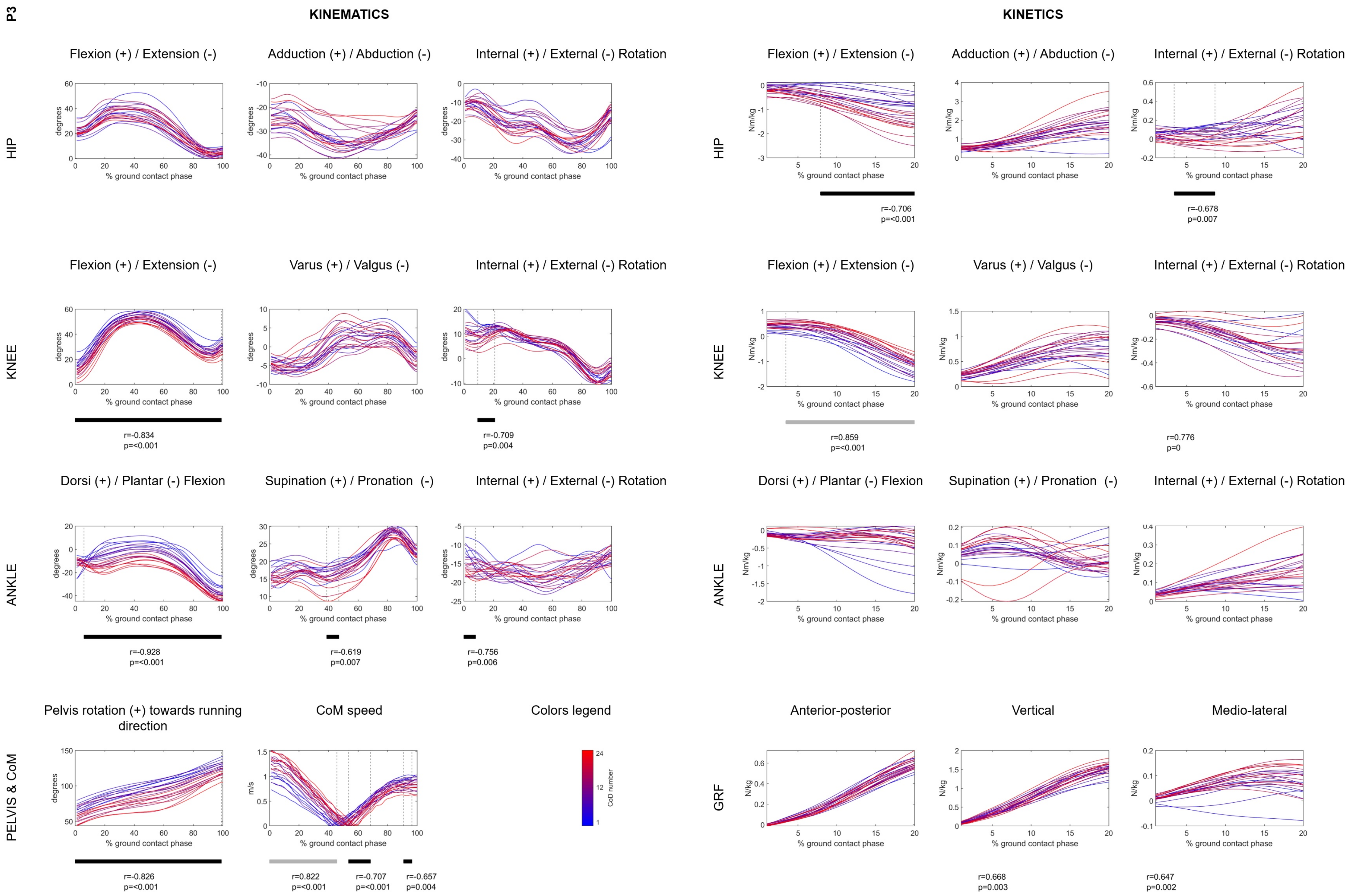
P3
KINEMATICS
KINETICS
HIP
Flexion (+) / Extension (-)
Adduction (+) / Abduction (-)
Internal (+) / External (-) Rotation
Flexion (+) / Extension (-)
Adduction (+) / Abduction (-)
Internal (+) / External (-) Rotation
r=-0.706
p=<0.001
r=-0.678
p=0.007
KNEE
Flexion (+) / Extension (-)
Varus (+) / Valgus (-)
Internal (+) / External (-) Rotation
Flexion (+) / Extension (-)
Varus (+) / Valgus (-)
Internal (+) / External (-) Rotation
r=-0.834
p=<0.001
r=-0.709
p=0.004
r=0.859
p=<0.001
r=0.776
p=0
ANKLE
Dorsi (+) / Plantar (-) Flexion
Supination (+) / Pronation (-)
Internal (+) / External (-) Rotation
Dorsi (+) / Plantar (-) Flexion
Supination (+) / Pronation (-)
Internal (+) / External (-) Rotation
r=-0.928
p=<0.001
r=-0.619
p=0.007
r=-0.756
p=0.006
PELVIS & CoM
Pelvis rotation (+) towards running direction
CoM speed
Colors legend
CoD number
r=-0.826
p=<0.001
r=0.822
p=<0.001
r=-0.707
p=<0.001
r=-0.657
p=0.004
GRF
Anterior-posterior
Vertical
Medio-lateral
r=0.668
p=0.003
r=0.647
p=0.002
degrees
Nm/kg
N/kg
m/s
% ground contact phase

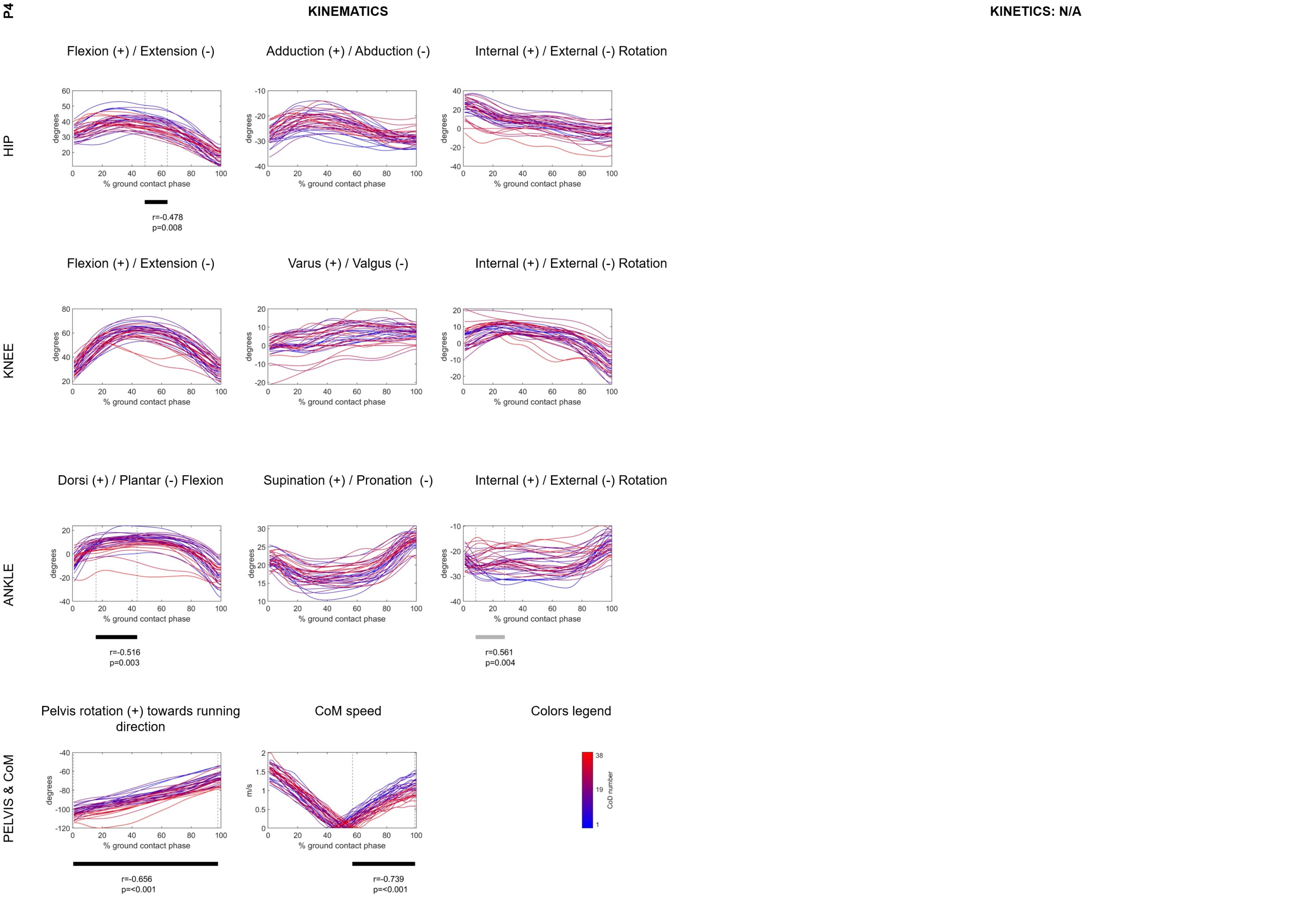
P4
KINEMATICS
KINETICS: N/A
HIP
Flexion (+) / Extension (-)
Adduction (+) / Abduction (-)
Internal (+) / External (-) Rotation
r=-0.478
p=0.008
KNEE
Flexion (+) / Extension (-)
Varus (+) / Valgus (-)
Internal (+) / External (-) Rotation
ANKLE
Dorsi (+) / Plantar (-) Flexion
Supination (+) / Pronation (-)
Internal (+) / External (-) Rotation
r=-0.516
p=0.003
r=0.561
p=0.004
PELVIS & CoM
Pelvis rotation (+) towards running direction
CoM speed
Colors legend
r=-0.656
p=<0.001
r=-0.739
p=<0.001
degrees
m/s
% ground contact phase
CoD number
38
19
1

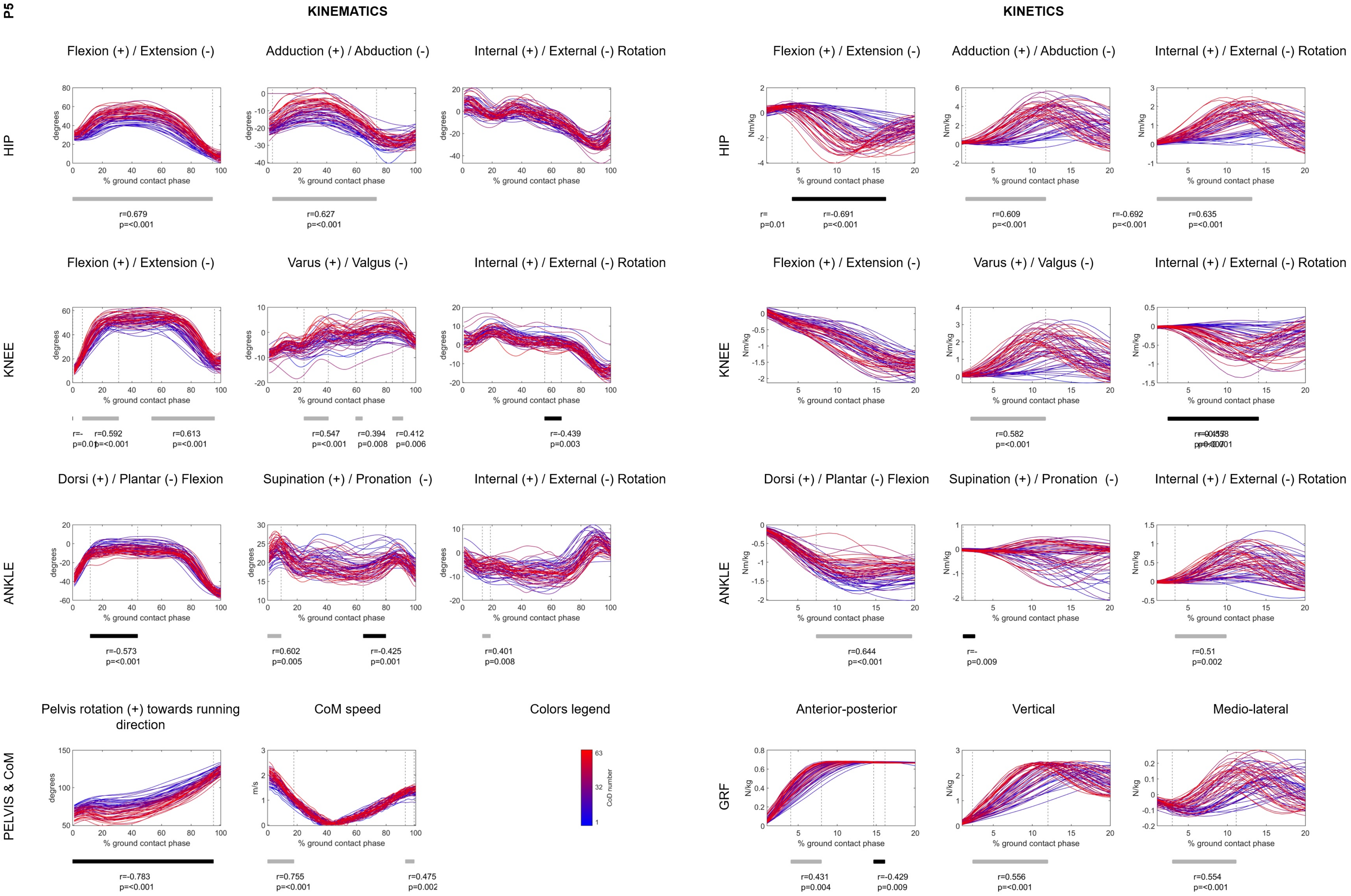

P5
KINEMATICS
KINETICS
HIP
KNEE
ANKLE
PELVIS & CoM
GRF
Flexion (+) / Extension (-)
Adduction (+) / Abduction (-)
Internal (+) / External (-) Rotation
Varus (+) / Valgus (-)
Dorsi (+) / Plantar (-) Flexion
Supination (+) / Pronation (-)
Pelvis rotation (+) towards running direction
CoM speed
Colors legend
Anterior-posterior
Vertical
Medio-lateral
degrees
Nm/kg
m/s
N/kg
% ground contact phase
CoD number
r=0.679 p=<0.001
r=0.627 p=<0.001
r= p=0.01
r=-0.691 p=<0.001
r=0.609 p=<0.001
r=-0.692 p=<0.001
r=0.635 p=<0.001
r=- p=0.01
r=0.592 p=<0.001
r=0.613 p=<0.001
r=0.547 p=<0.001
r=0.394 p=0.008
r=0.412 p=0.006
r=-0.439 p=0.003
r=0.582 p=<0.001
r=-0.573 p=<0.001
r=0.602 p=0.005
r=-0.425 p=0.001
r=0.401 p=0.008
r=0.644 p=<0.001
r=- p=0.009
r=0.51 p=0.002
r=-0.783 p=<0.001
r=0.755 p=<0.001
r=0.475 p=0.002
r=0.431 p=0.004
r=-0.429 p=0.009
r=0.556 p=<0.001
r=0.554 p=<0.001

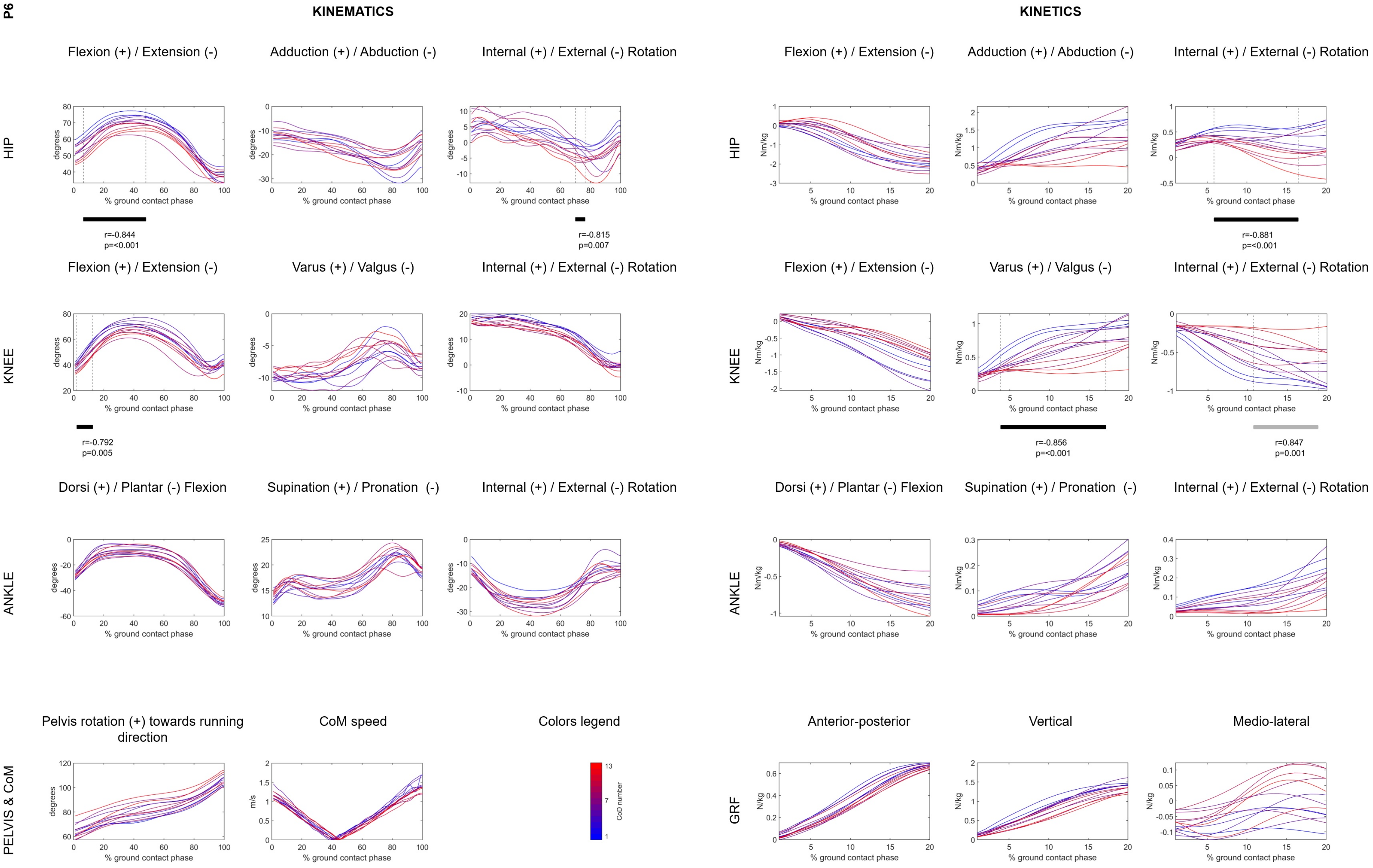

P6
KINEMATICS
KINETICS
HIP
Flexion (+) / Extension (-)
Adduction (+) / Abduction (-)
Internal (+) / External (-) Rotation
Flexion (+) / Extension (-)
Adduction (+) / Abduction (-)
Internal (+) / External (-) Rotation
r=-0.844
p=<0.001
r=-0.815
p=0.007
r=-0.881
p=<0.001
KNEE
Flexion (+) / Extension (-)
Varus (+) / Valgus (-)
Internal (+) / External (-) Rotation
Flexion (+) / Extension (-)
Varus (+) / Valgus (-)
Internal (+) / External (-) Rotation
r=-0.792
p=0.005
r=-0.856
p=<0.001
r=0.847
p=0.001
ANKLE
Dorsi (+) / Plantar (-) Flexion
Supination (+) / Pronation (-)
Internal (+) / External (-) Rotation
Dorsi (+) / Plantar (-) Flexion
Supination (+) / Pronation (-)
Internal (+) / External (-) Rotation
PELVIS & CoM
Pelvis rotation (+) towards running direction
CoM speed
Colors legend
CoD number
GRF
Anterior-posterior
Vertical
Medio-lateral
degrees
Nm/kg
m/s
N/kg
% ground contact phase

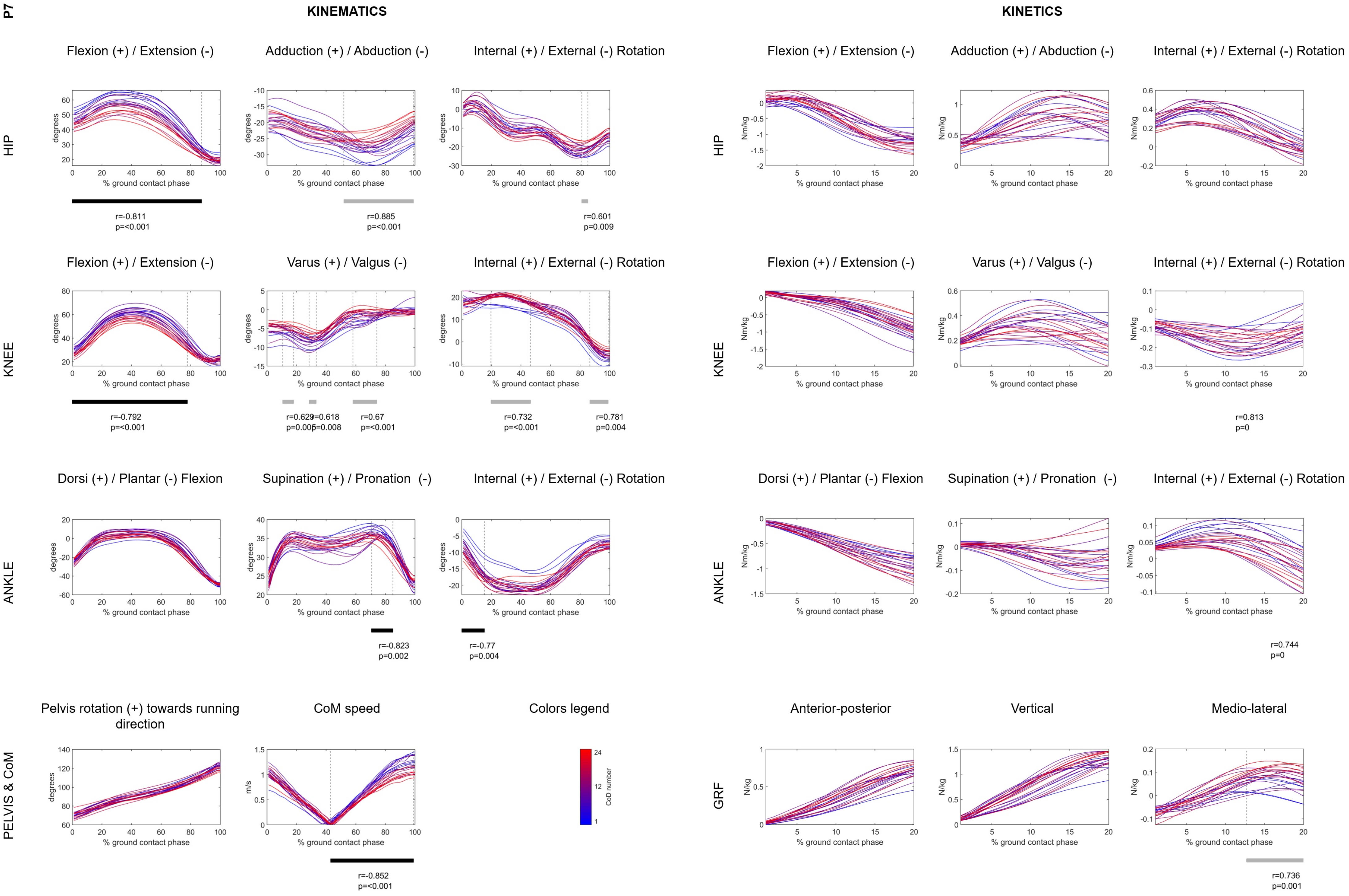

P7
KINEMATICS
KINETICS
HIP
Flexion (+) / Extension (-)
Adduction (+) / Abduction (-)
Internal (+) / External (-) Rotation
r=-0.811
p=<0.001
r=0.885
p=<0.001
r=0.601
p=0.009
KNEE
Flexion (+) / Extension (-)
Varus (+) / Valgus (-)
Internal (+) / External (-) Rotation
r=-0.792
p=<0.001
r=0.629
r=0.618
p=0.008
r=0.67
p=<0.001
r=0.732
p=<0.001
r=0.781
p=0.004
r=0.813
p=0
ANKLE
Dorsi (+) / Plantar (-) Flexion
Supination (+) / Pronation (-)
Internal (+) / External (-) Rotation
r=-0.823
p=0.002
r=-0.77
p=0.004
r=0.744
p=0
PELVIS & CoM
Pelvis rotation (+) towards running direction
CoM speed
Colors legend
CoD number
r=-0.852
p=<0.001
GRF
Anterior-posterior
Vertical
Medio-lateral
r=0.736
p=0.001
degrees
Nm/kg
N/kg
m/s
% ground contact phase

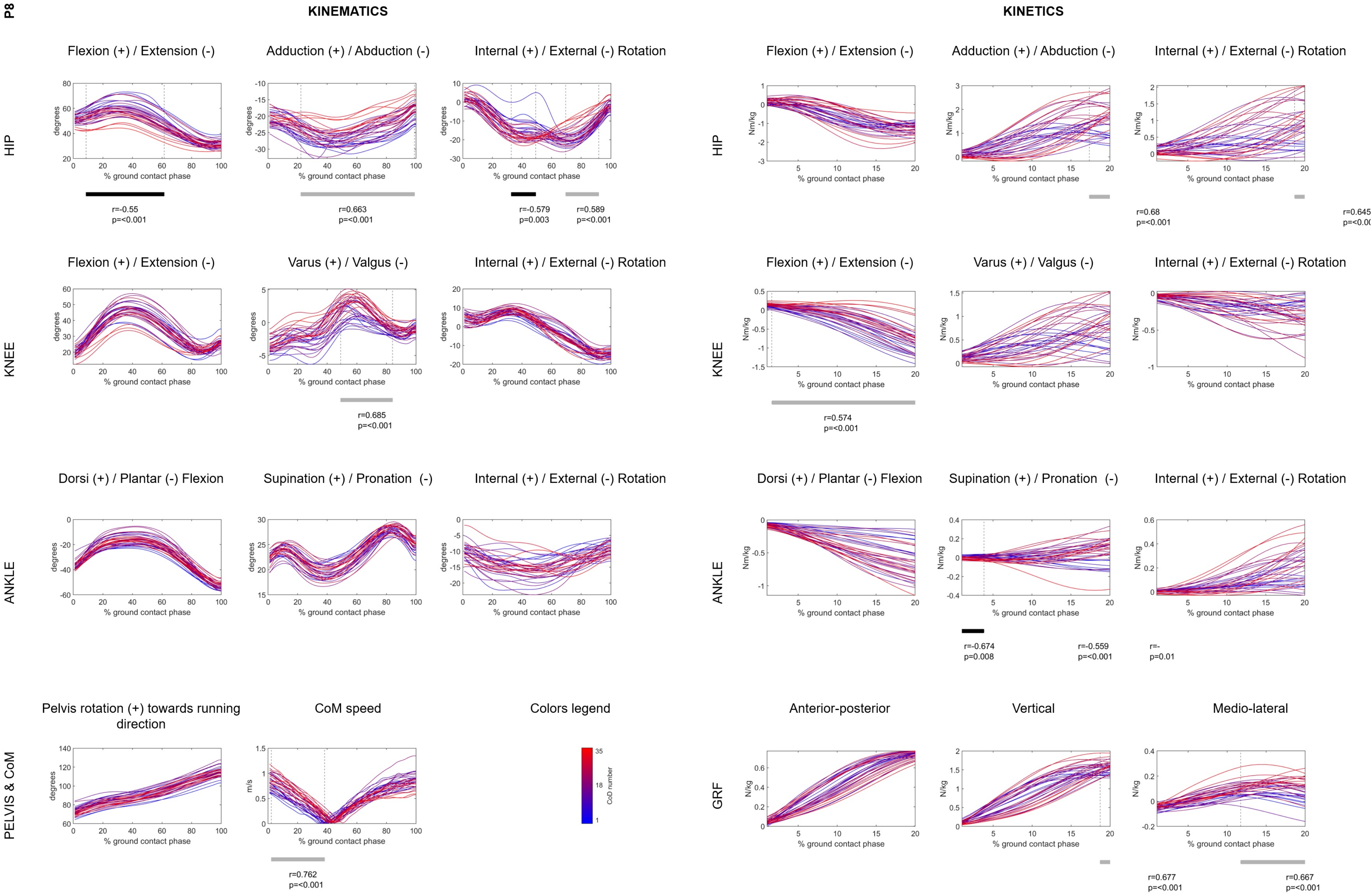
P8
KINEMATICS
KINETICS
HIP
KNEE
ANKLE
PELVIS & CoM
GRF
Flexion (+) / Extension (-)
Adduction (+) / Abduction (-)
Internal (+) / External (-) Rotation
Varus (+) / Valgus (-)
Dorsi (+) / Plantar (-) Flexion
Supination (+) / Pronation (-)
Pelvis rotation (+) towards running direction
CoM speed
Colors legend
Anterior-posterior
Vertical
Medio-lateral
degrees
Nm/kg
m/s
N/kg
% ground contact phase
CoD number
r=-0.55
p=<0.001
r=0.663
p=<0.001
r=-0.579
p=0.003
r=0.589
p=<0.001
r=0.68
p=<0.001
r=0.645
r=0.685
p=<0.001
r=0.574
p=<0.001
r=-0.674
p=0.008
r=-0.559
p=<0.001
r=-
p=0.01
r=0.762
p=<0.001
r=0.677
p=<0.001
r=0.667
p=<0.001

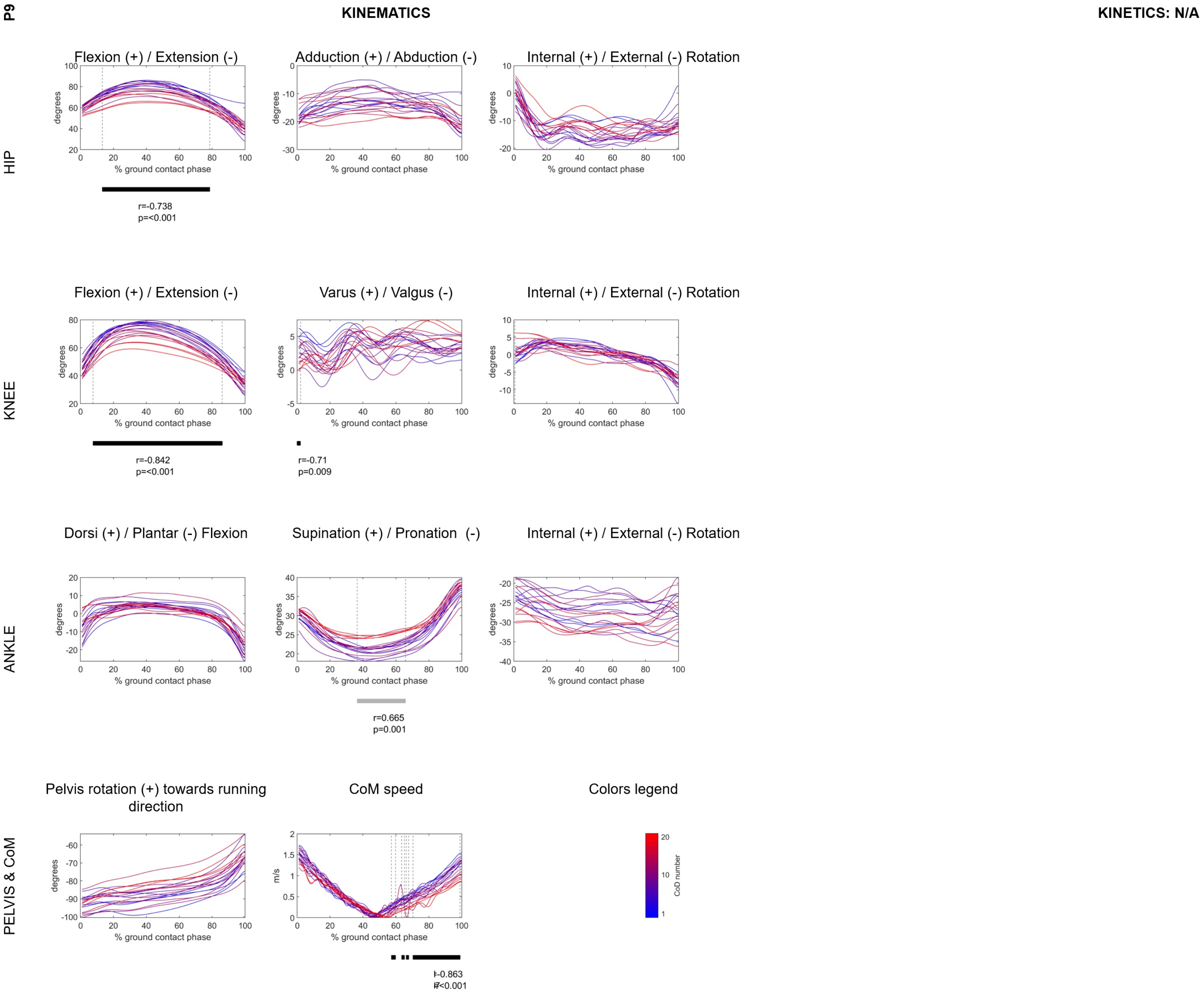
P9
KINEMATICS
KINETICS: N/A
HIP
Flexion (+) / Extension (-)
Adduction (+) / Abduction (-)
Internal (+) / External (-) Rotation
degrees
% ground contact phase
r=-0.738
p=<0.001
KNEE
Flexion (+) / Extension (-)
Varus (+) / Valgus (-)
Internal (+) / External (-) Rotation
r=-0.842
p=<0.001
r=-0.71
p=0.009
ANKLE
Dorsi (+) / Plantar (-) Flexion
Supination (+) / Pronation (-)
Internal (+) / External (-) Rotation
r=0.665
p=0.001
PELVIS & CoM
Pelvis rotation (+) towards running direction
CoM speed
m/s
Colors legend
CoD number
r=-0.863
p=<0.001

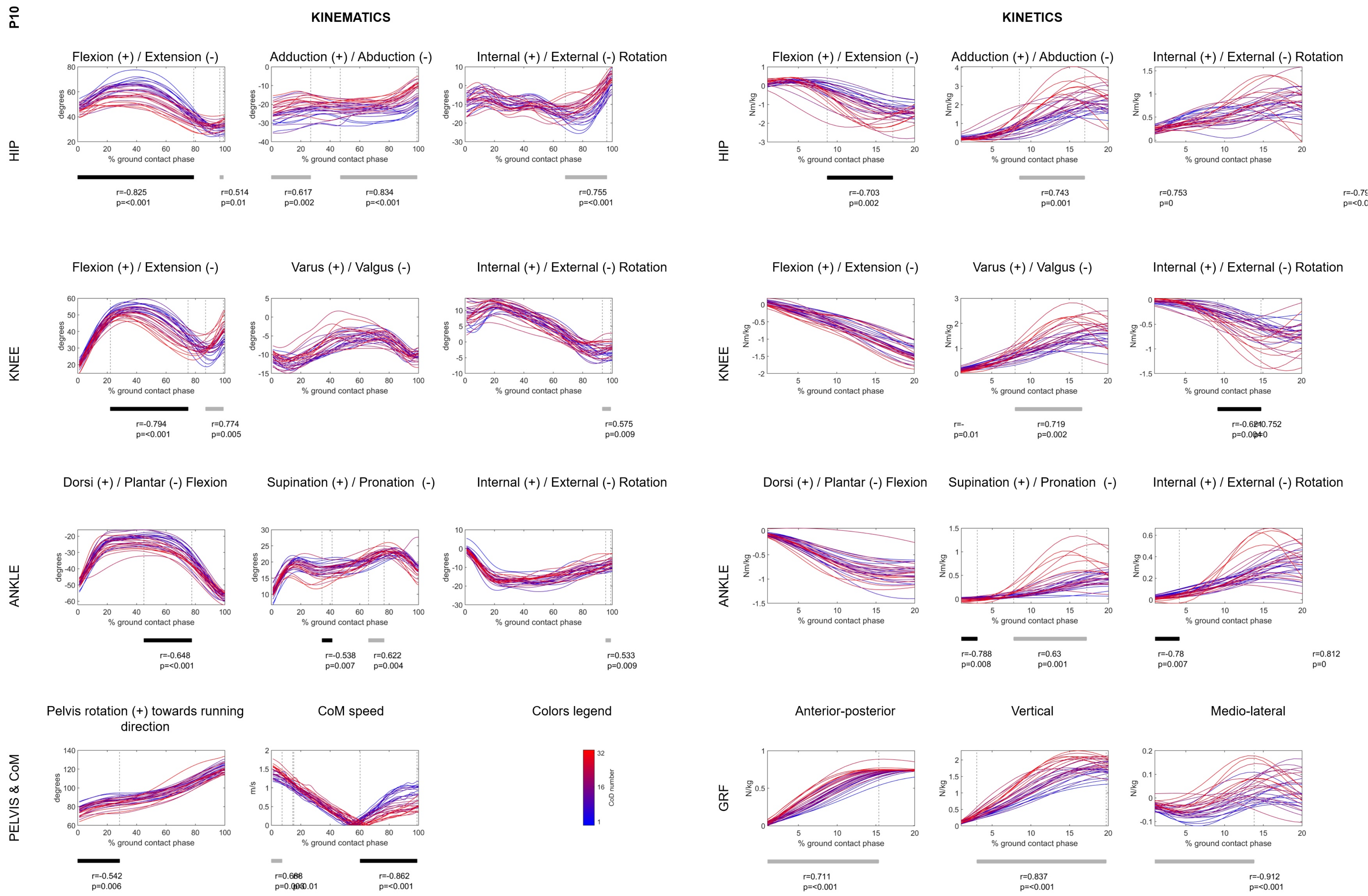
P10
KINEMATICS
KINETICS
HIP
Flexion (+) / Extension (-)
Adduction (+) / Abduction (-)
Internal (+) / External (-) Rotation
degrees
Nm/kg
% ground contact phase
r=-0.825
p=<0.001
r=0.514
p=0.01
r=0.617
p=0.002
r=0.834
p=<0.001
r=0.755
p=<0.001
r=-0.703
p=0.002
r=0.743
p=0.001
r=0.753
p=0
KNEE
Flexion (+) / Extension (-)
Varus (+) / Valgus (-)
Internal (+) / External (-) Rotation
r=-0.794
p=<0.001
r=0.774
p=0.005
r=0.575
p=0.009
r=-
p=0.01
r=0.719
p=0.002
ANKLE
Dorsi (+) / Plantar (-) Flexion
Supination (+) / Pronation (-)
Internal (+) / External (-) Rotation
r=-0.648
p=<0.001
r=-0.538
p=0.007
r=0.622
p=0.004
r=0.533
p=0.009
r=-0.788
p=0.008
r=0.63
p=0.001
r=-0.78
p=0.007
r=0.812
p=0
PELVIS & CoM
Pelvis rotation (+) towards running direction
CoM speed
m/s
Colors legend
CoD number
r=-0.542
p=0.006
r=-0.862
p=<0.001
GRF
Anterior-posterior
Vertical
Medio-lateral
N/kg
r=0.711
p=<0.001
r=0.837
p=<0.001
r=-0.912
p=<0.001

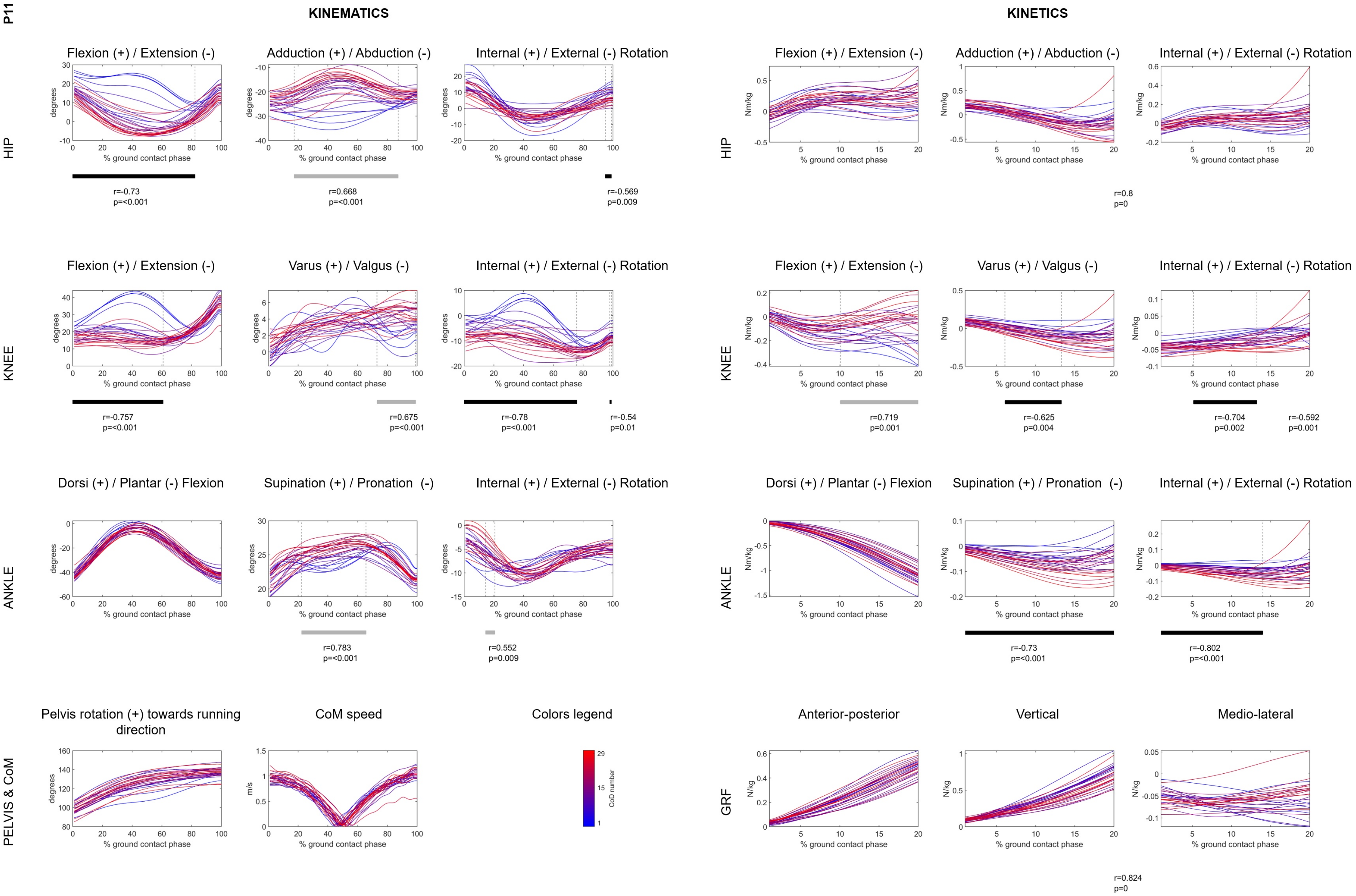
P11
KINEMATICS
KINETICS
HIP
KNEE
ANKLE
PELVIS & CoM
GRF
Flexion (+) / Extension (-)
Adduction (+) / Abduction (-)
Internal (+) / External (-) Rotation
Varus (+) / Valgus (-)
Dorsi (+) / Plantar (-) Flexion
Supination (+) / Pronation (-)
Pelvis rotation (+) towards running direction
CoM speed
Colors legend
Anterior-posterior
Vertical
Medio-lateral
degrees
Nm/kg
N/kg
m/s
% ground contact phase
CoD number
r=-0.73
p=<0.001
r=0.668
p=<0.001
r=-0.569
p=0.009
r=0.8
p=0
r=-0.757
p=<0.001
r=0.675
p=<0.001
r=-0.78
p=<0.001
r=-0.54
p=0.01
r=0.719
p=0.001
r=-0.625
p=0.004
r=-0.704
p=0.002
r=-0.592
p=0.001
r=0.783
p=<0.001
r=0.552
p=0.009
r=-0.73
p=<0.001
r=-0.802
p=<0.001
r=0.824
p=0

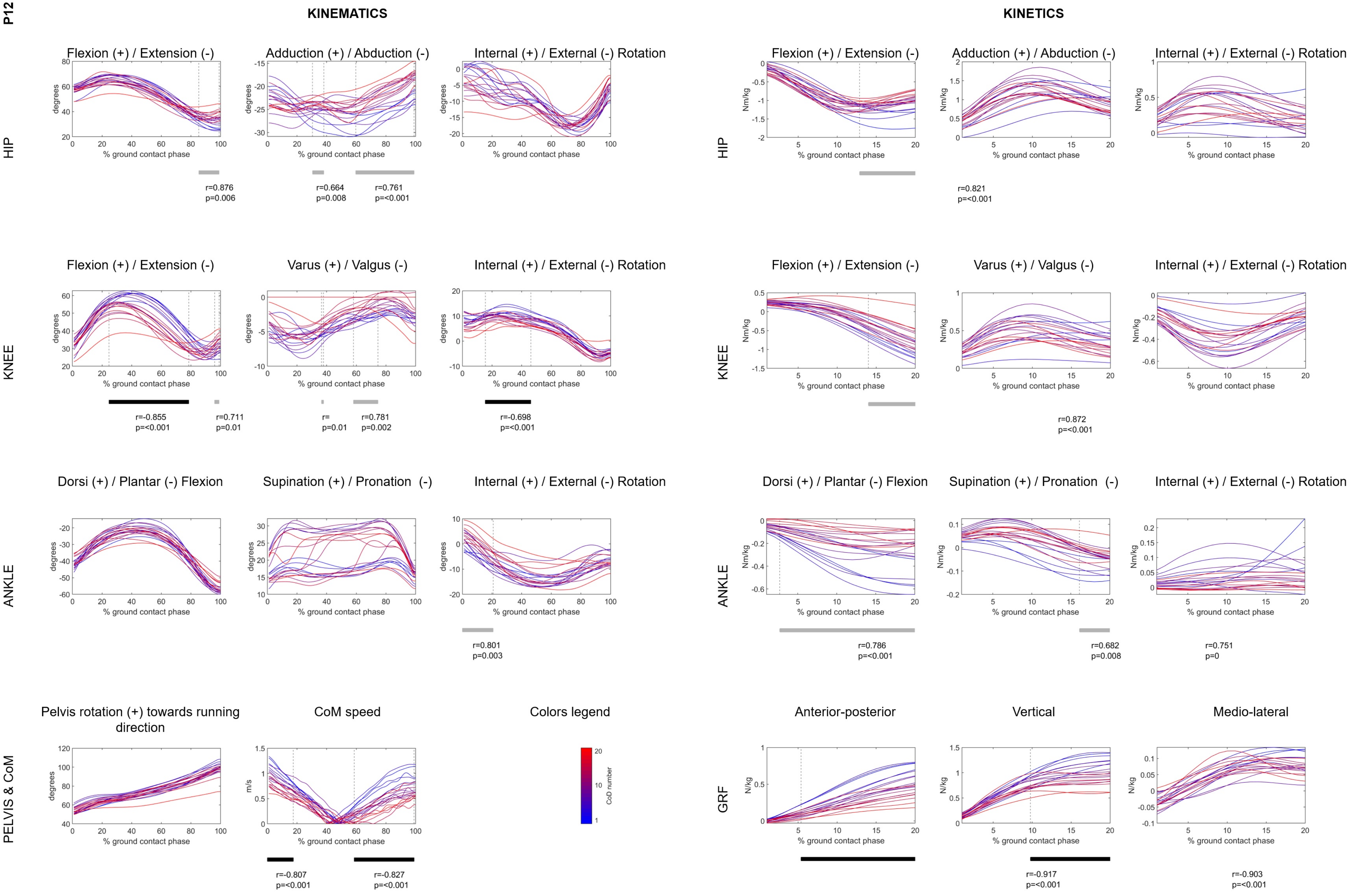
P12
KINEMATICS
KINETICS
HIP
KNEE
ANKLE
PELVIS & CoM
GRF
Flexion (+) / Extension (-)
Adduction (+) / Abduction (-)
Internal (+) / External (-) Rotation
Varus (+) / Valgus (-)
Dorsi (+) / Plantar (-) Flexion
Supination (+) / Pronation (-)
Pelvis rotation (+) towards running direction
CoM speed
Colors legend
Anterior-posterior
Vertical
Medio-lateral
degrees
Nm/kg
m/s
N/kg
% ground contact phase
CoD number
r=0.876
p=0.006
r=0.664
p=0.008
r=0.761
p=<0.001
r=0.821
p=<0.001
r=-0.855
p=<0.001
r=0.711
p=0.01
r=
p=0.01
r=0.781
p=0.002
r=-0.698
p=<0.001
r=0.872
p=<0.001
r=0.801
p=0.003
r=0.786
p=<0.001
r=0.682
p=0.008
r=0.751
p=0
r=-0.807
p=<0.001
r=-0.827
p=<0.001
r=-0.917
p=<0.001
r=-0.903
p=<0.001

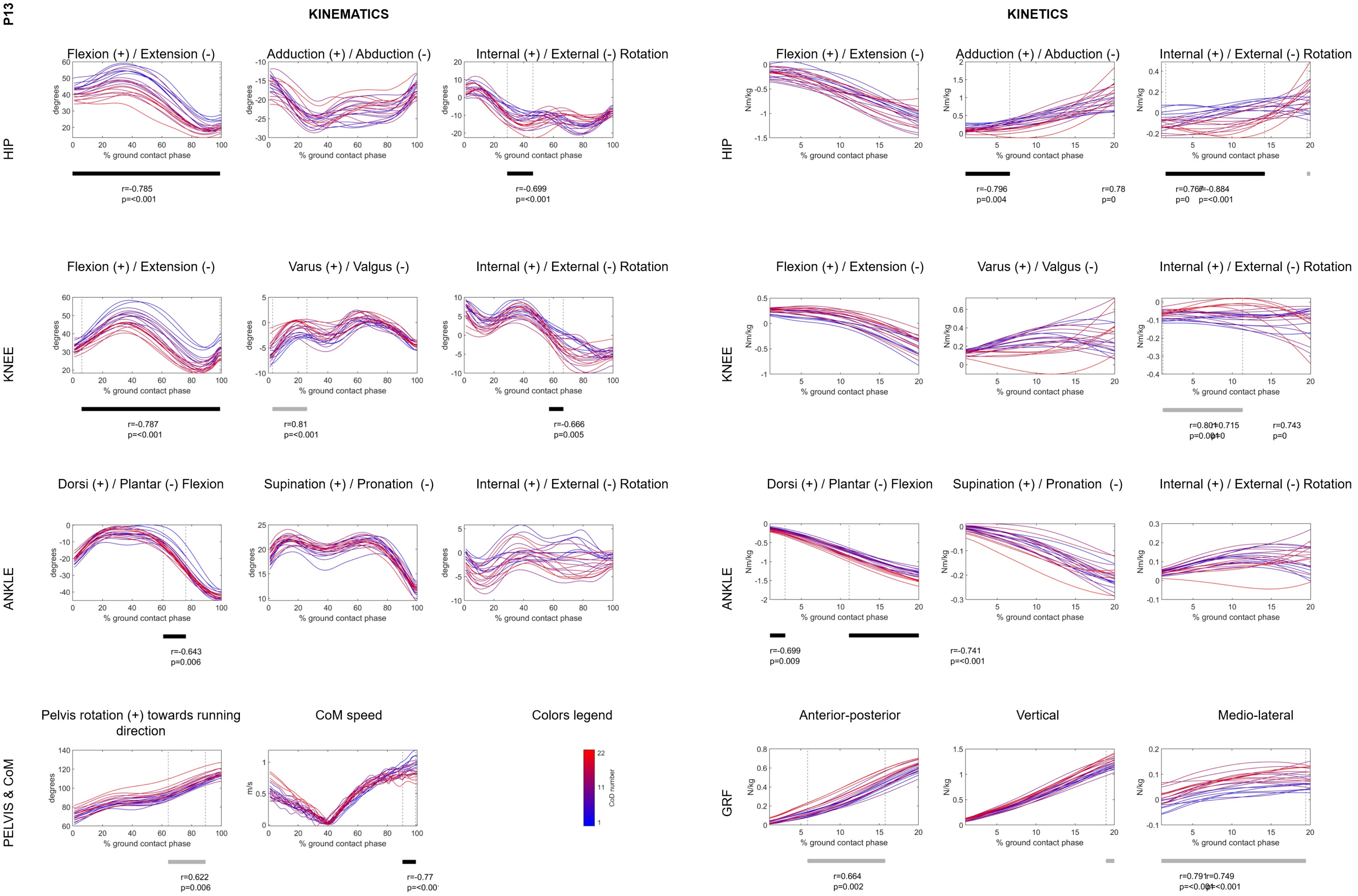
P13
KINEMATICS
KINETICS
HIP
KNEE
ANKLE
PELVIS & CoM
GRF
Flexion (+) / Extension (-)
Adduction (+) / Abduction (-)
Internal (+) / External (-) Rotation
Varus (+) / Valgus (-)
Dorsi (+) / Plantar (-) Flexion
Supination (+) / Pronation (-)
Pelvis rotation (+) towards running direction
CoM speed
Colors legend
Anterior-posterior
Vertical
Medio-lateral
degrees
Nm/kg
m/s
N/kg
% ground contact phase
CoD number
r=-0.785
p=<0.001
r=-0.699
p=<0.001
r=-0.796
p=0.004
r=0.78
p=0
r=0.767
p=0
r=-0.884
p=<0.001
r=-0.787
p=<0.001
r=0.81
p=<0.001
r=-0.666
p=0.005
r=0.801
r=0.715
r=0.743
p=0
r=-0.643
p=0.006
r=-0.699
p=0.009
r=-0.741
p=<0.001
r=0.622
p=0.006
r=-0.77
r=0.664
p=0.002
r=0.791
r=0.749
p=<0.001

P14

KINEMATICS

KINETICS: N/A

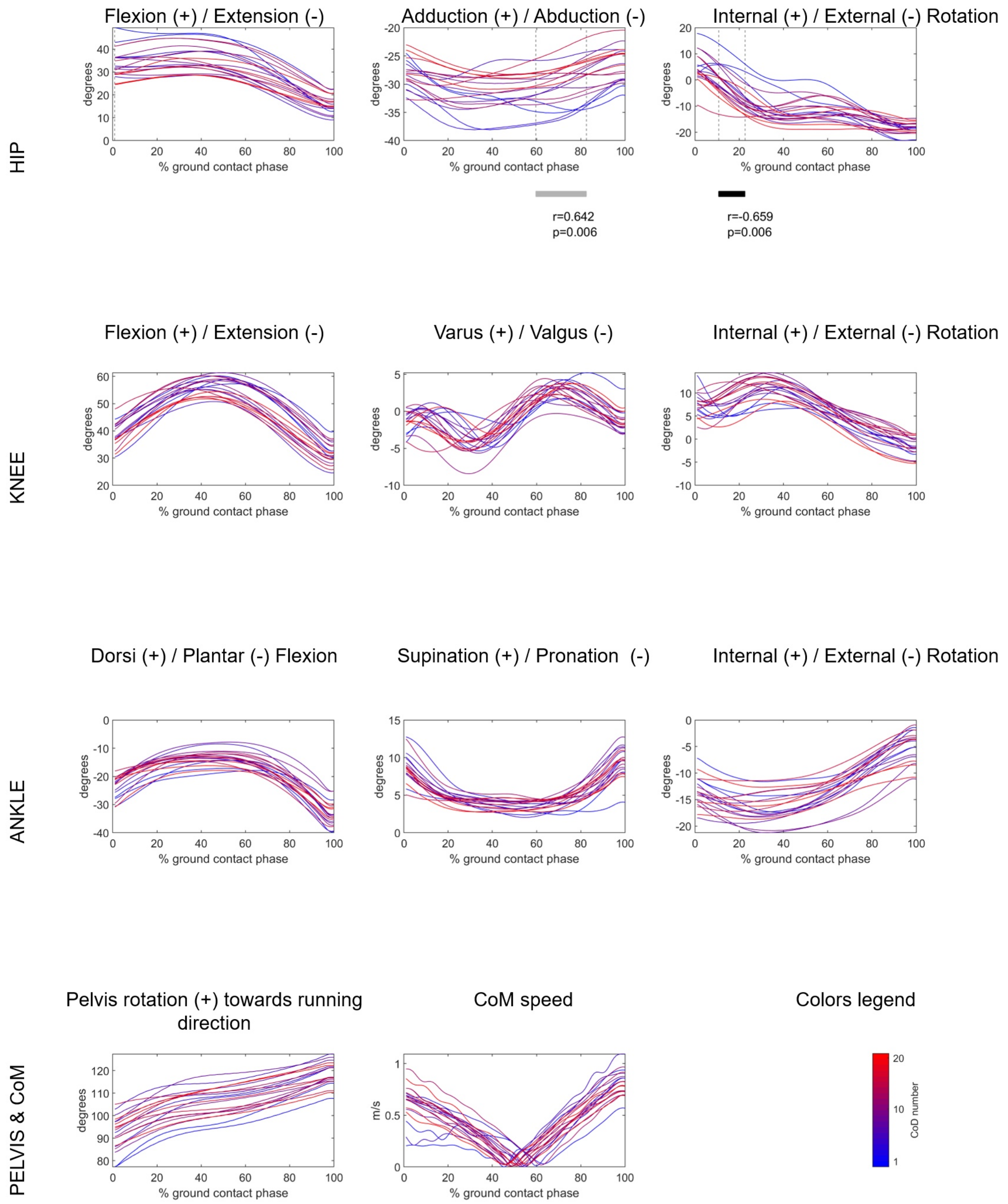

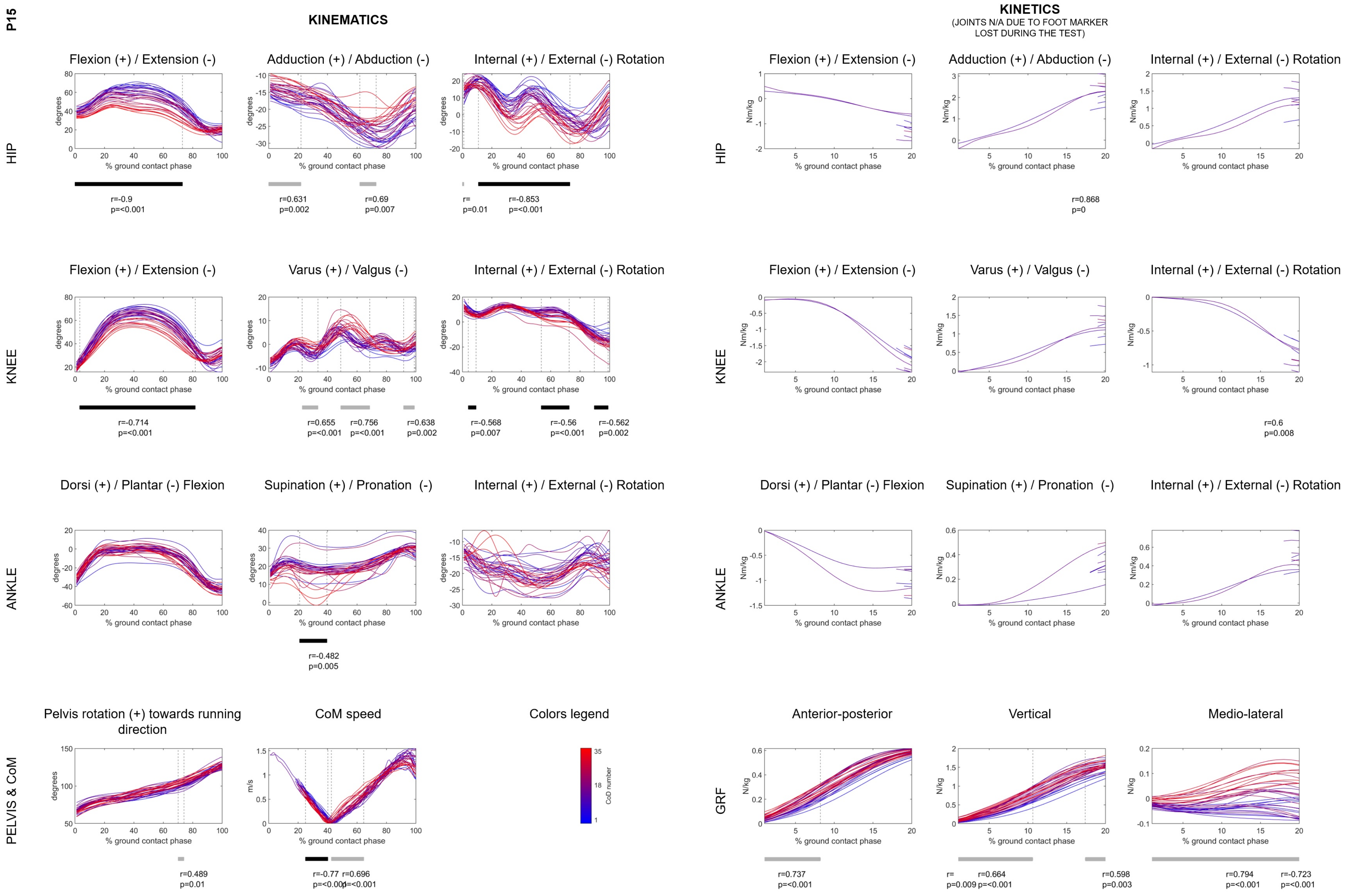
P15
KINEMATICS
KINETICS
(JOINTS N/A DUE TO FOOT MARKER
LOST DURING THE TEST)
HIP
KNEE
ANKLE
PELVIS & CoM
GRF
Flexion (+) / Extension (-)
Adduction (+) / Abduction (-)
Internal (+) / External (-) Rotation
Varus (+) / Valgus (-)
Dorsi (+) / Plantar (-) Flexion
Supination (+) / Pronation (-)
Pelvis rotation (+) towards running direction
CoM speed
Colors legend
Anterior-posterior
Vertical
Medio-lateral
degrees
Nm/kg
N/kg
m/s
% ground contact phase
CoD number
r=-0.9
p=<0.001
r=0.631
p=0.002
r=0.69
p=0.007
r=
p=0.01
r=-0.853
p=<0.001
r=0.868
p=0
r=-0.714
p=<0.001
r=0.655
p=<0.001
r=0.756
p=<0.001
r=0.638
p=0.002
r=-0.568
p=0.007
r=-0.56
p=<0.001
r=-0.562
p=0.002
r=0.6
p=0.008
r=-0.482
p=0.005
r=0.489
p=0.01
r=-0.77
p=<0.001
r=0.696
p=<0.001
r=0.737
p=<0.001
r=
p=0.009
r=0.664
p=<0.001
r=0.598
p=0.003
r=0.794
p=<0.001
r=-0.723
p=<0.001

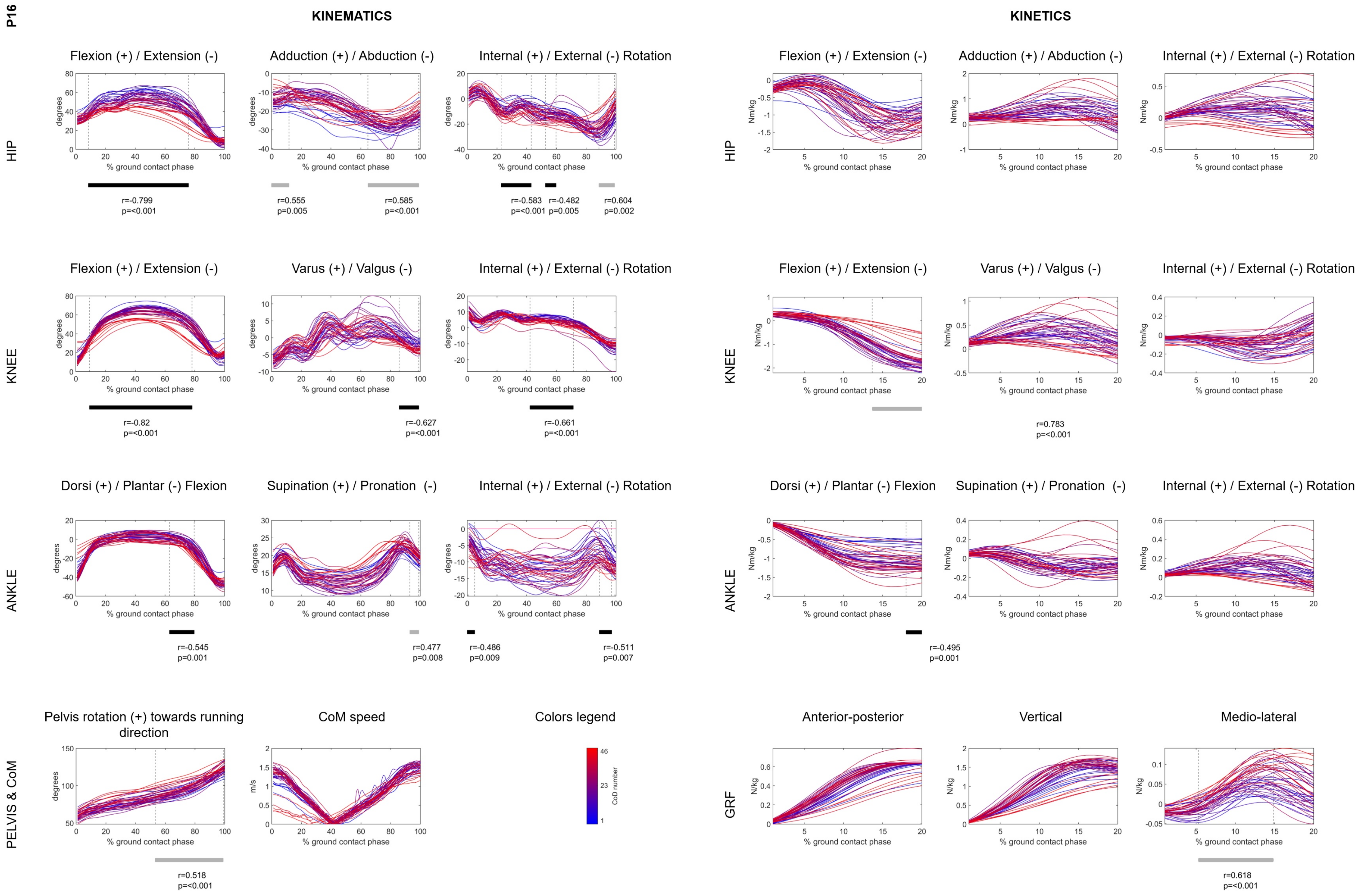
P16
KINEMATICS
KINETICS
HIP
KNEE
ANKLE
PELVIS & CoM
GRF
Flexion (+) / Extension (-)
Adduction (+) / Abduction (-)
Internal (+) / External (-) Rotation
Varus (+) / Valgus (-)
Dorsi (+) / Plantar (-) Flexion
Supination (+) / Pronation (-)
Pelvis rotation (+) towards running direction
CoM speed
Colors legend
Anterior-posterior
Vertical
Medio-lateral
degrees
Nm/kg
m/s
N/kg
% ground contact phase
CoD number
r=-0.799 p=<0.001
r=0.555 p=0.005
r=0.585 p=<0.001
r=-0.583 p=<0.001
r=-0.482 p=0.005
r=0.604 p=0.002
r=-0.82 p=<0.001
r=-0.627 p=<0.001
r=-0.661 p=<0.001
r=0.783 p=<0.001
r=-0.545 p=0.001
r=0.477 p=0.008
r=-0.486 p=0.009
r=-0.511 p=0.007
r=-0.495 p=0.001
r=0.518 p=<0.001
r=0.618 p=<0.001

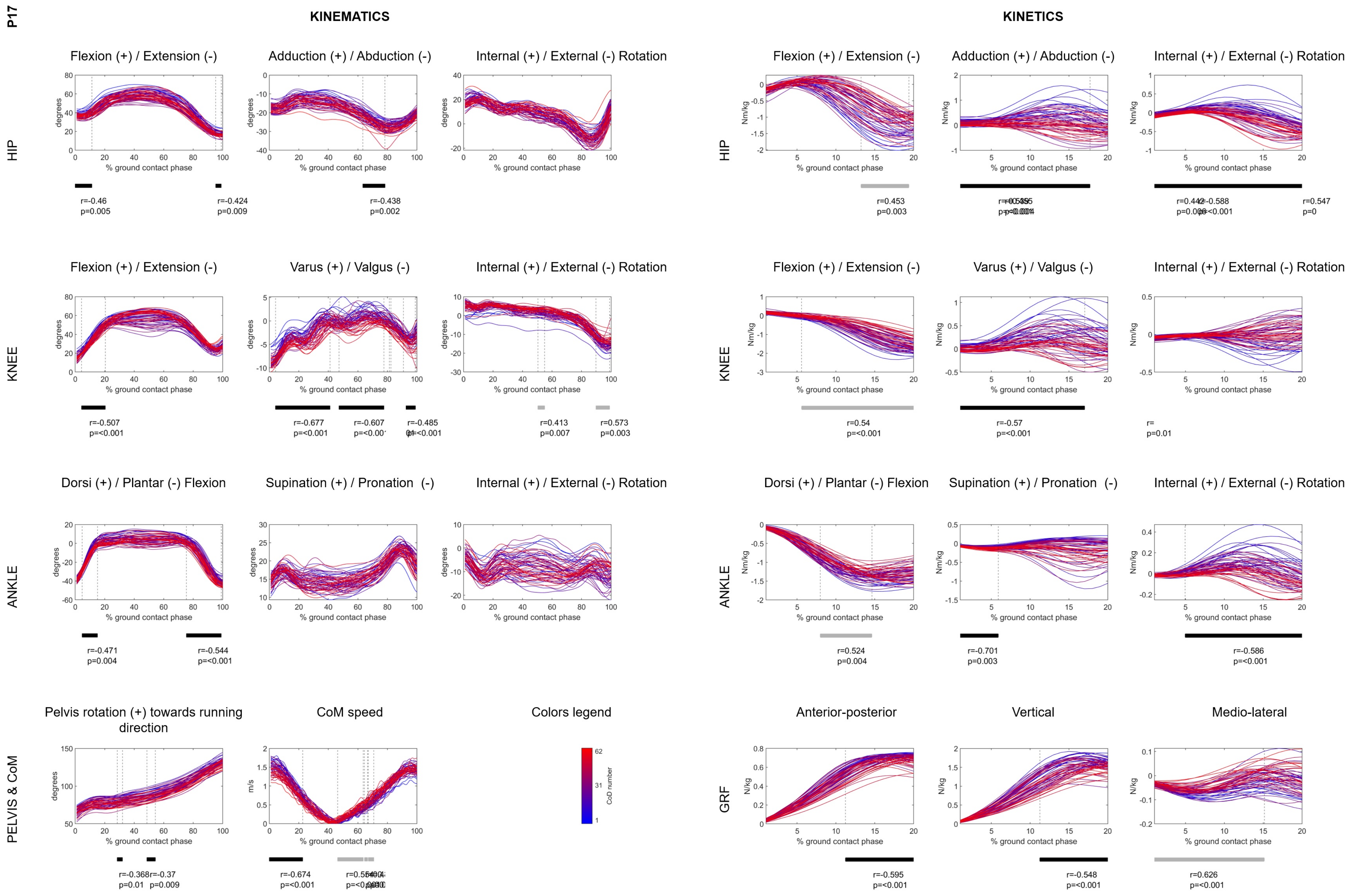
P17
KINEMATICS
KINETICS
HIP
KNEE
ANKLE
PELVIS & CoM
GRF
Flexion (+) / Extension (-)
Adduction (+) / Abduction (-)
Internal (+) / External (-) Rotation
Varus (+) / Valgus (-)
Dorsi (+) / Plantar (-) Flexion
Supination (+) / Pronation (-)
Pelvis rotation (+) towards running direction
CoM speed
Colors legend
CoD number
Anterior-posterior
Vertical
Medio-lateral
degrees
Nm/kg
N/kg
m/s
% ground contact phase
r=-0.46
p=0.005
r=-0.424
p=0.009
r=-0.438
p=0.002
r=0.453
p=0.003
r=0.547
p=0
r=-0.507
p=<0.001
r=-0.677
p=<0.001
r=-0.485
r=0.413
p=0.007
r=0.573
p=0.003
r=0.54
p=<0.001
r=-0.57
p=<0.001
r=
p=0.01
r=-0.471
p=0.004
r=-0.544
p=<0.001
r=0.524
p=0.004
r=-0.701
p=0.003
r=-0.586
p=<0.001
r=-0.368
p=0.01
r=-0.37
p=0.009
r=-0.674
p=<0.001
r=-0.595
p=<0.001
r=-0.548
p=<0.001
r=0.626
p=<0.001

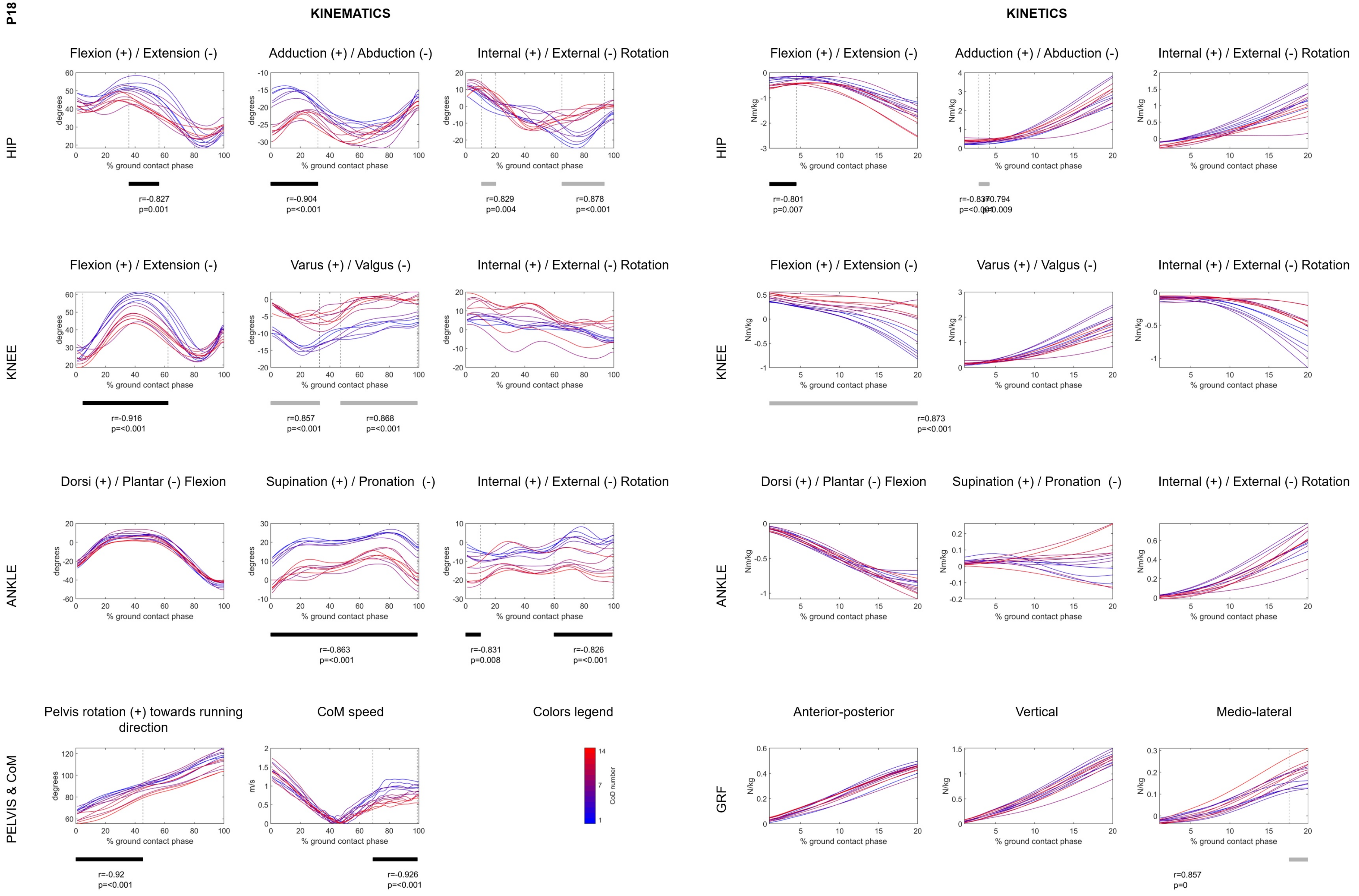

P18
KINEMATICS
KINETICS
HIP
KNEE
ANKLE
PELVIS & CoM
GRF
Flexion (+) / Extension (-)
Adduction (+) / Abduction (-)
Internal (+) / External (-) Rotation
Varus (+) / Valgus (-)
Dorsi (+) / Plantar (-) Flexion
Supination (+) / Pronation (-)
Pelvis rotation (+) towards running direction
CoM speed
Colors legend
Anterior-posterior
Vertical
Medio-lateral
degrees
Nm/kg
m/s
N/kg
% ground contact phase
CoD number
r=-0.827
p=0.001
r=-0.904
p=<0.001
r=0.829
p=0.004
r=0.878
p=<0.001
r=-0.801
p=0.007
r=-0.837
p=<0.001
r=0.794
p=0.009
r=-0.916
p=<0.001
r=0.857
p=<0.001
r=0.868
p=<0.001
r=0.873
p=<0.001
r=-0.863
p=<0.001
r=-0.831
p=0.008
r=-0.826
p=<0.001
r=-0.92
p=<0.001
r=-0.926
p=<0.001
r=0.857
p=0

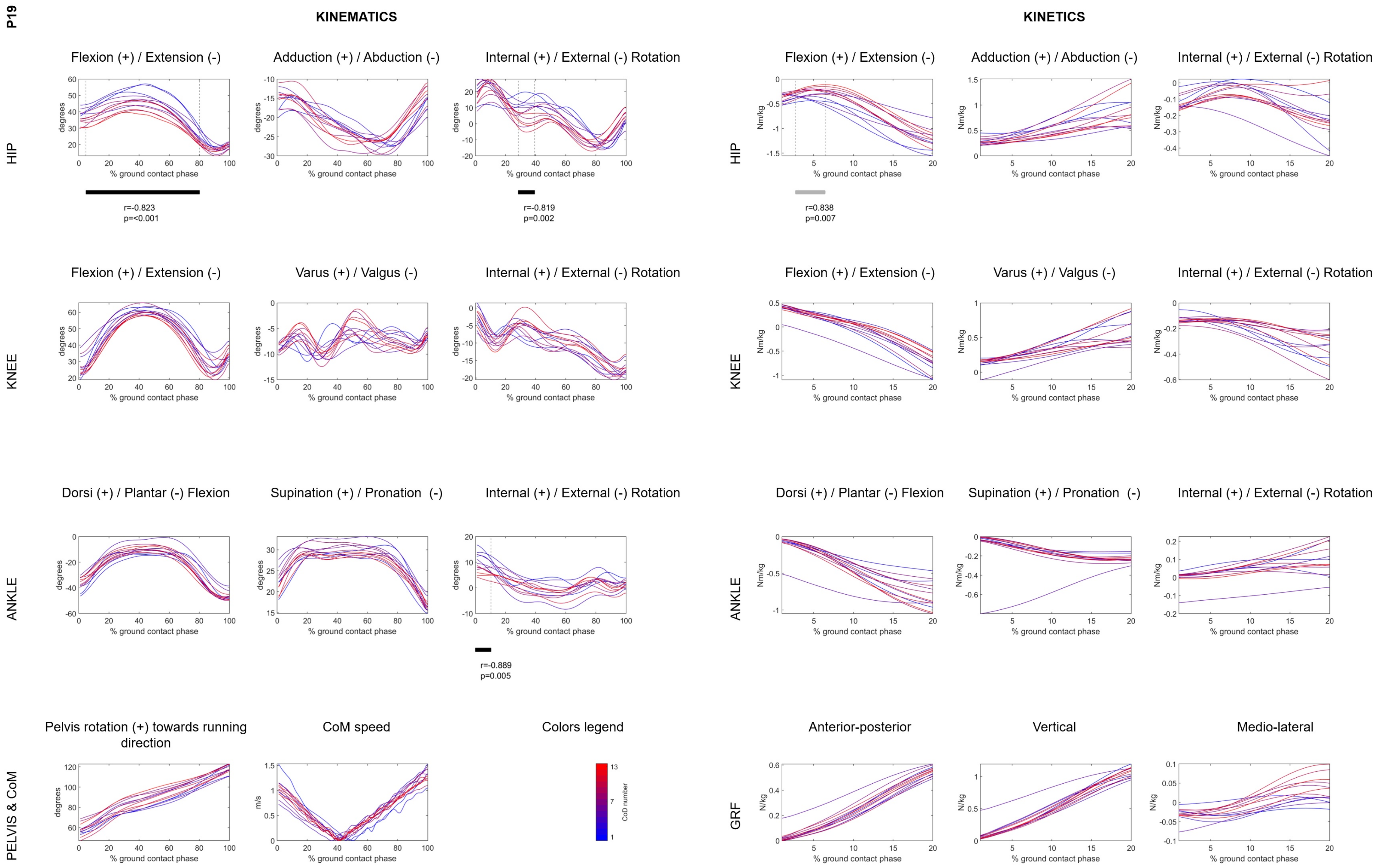
P19
KINEMATICS
KINETICS
HIP
Flexion (+) / Extension (-)
Adduction (+) / Abduction (-)
Internal (+) / External (-) Rotation
r=-0.823
p=<0.001
r=-0.819
p=0.002
r=0.838
p=0.007
KNEE
Flexion (+) / Extension (-)
Varus (+) / Valgus (-)
Internal (+) / External (-) Rotation
ANKLE
Dorsi (+) / Plantar (-) Flexion
Supination (+) / Pronation (-)
Internal (+) / External (-) Rotation
r=-0.889
p=0.005
PELVIS & CoM
Pelvis rotation (+) towards running direction
CoM speed
Colors legend
CoD number
GRF
Anterior-posterior
Vertical
Medio-lateral
degrees
Nm/kg
m/s
N/kg
% ground contact phase

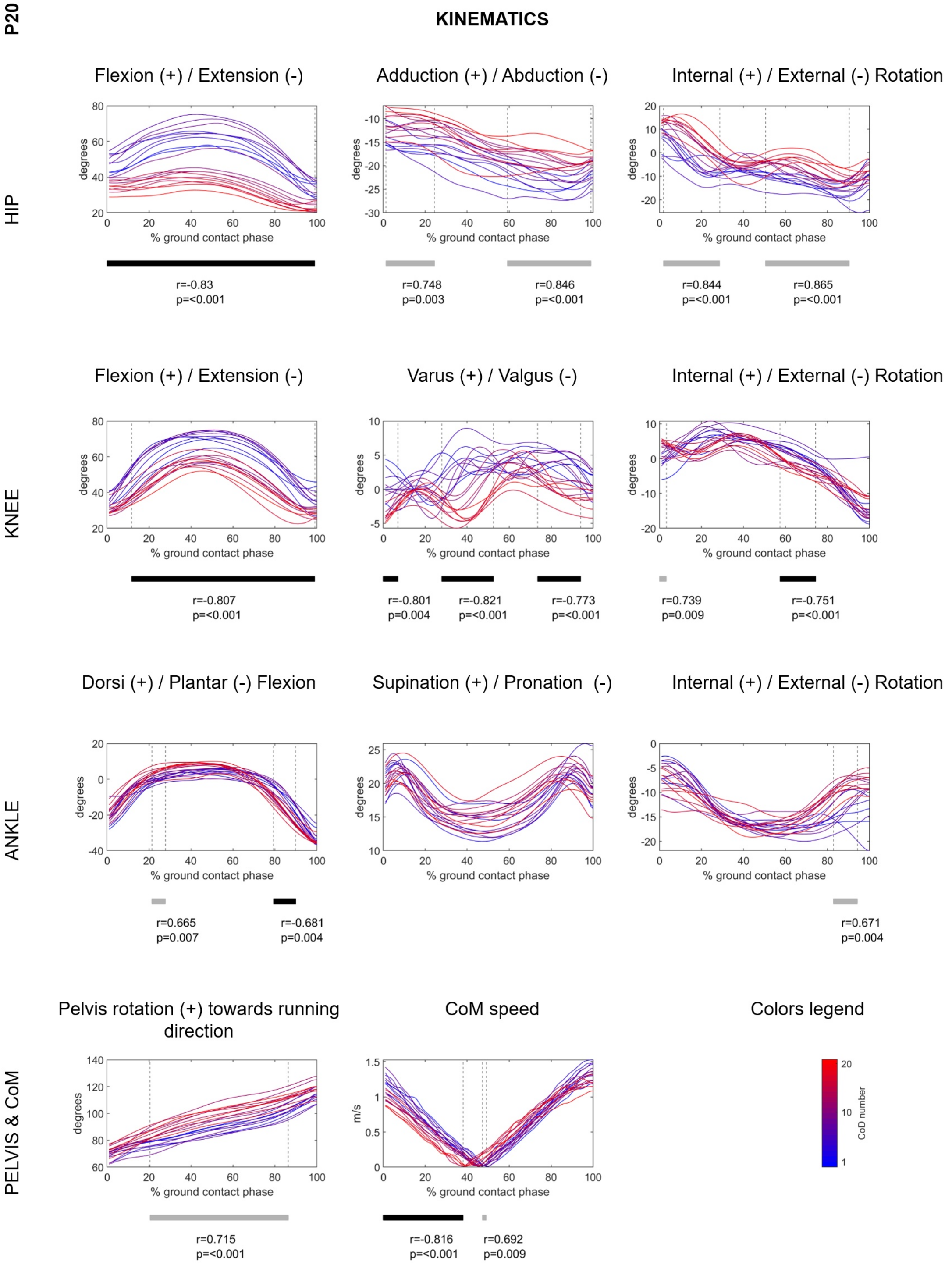
P20
KINEMATICS
KINETICS: N/A
HIP
Flexion (+) / Extension (-)
r=-0.83
p=<0.001
Adduction (+) / Abduction (-)
r=0.748
p=0.003
r=0.846
p=<0.001
Internal (+) / External (-) Rotation
r=0.844
p=<0.001
r=0.865
p=<0.001
KNEE
Flexion (+) / Extension (-)
r=-0.807
p=<0.001
Varus (+) / Valgus (-)
r=-0.801
p=0.004
r=-0.821
p=<0.001
r=-0.773
p=<0.001
Internal (+) / External (-) Rotation
r=0.739
p=0.009
r=-0.751
p=<0.001
ANKLE
Dorsi (+) / Plantar (-) Flexion
r=0.665
p=0.007
r=-0.681
p=0.004
Supination (+) / Pronation (-)
Internal (+) / External (-) Rotation
r=0.671
p=0.004
PELVIS & CoM
Pelvis rotation (+) towards running direction
r=0.715
p=<0.001
CoM speed
r=-0.816
p=<0.001
r=0.692
p=0.009
Colors legend
CoD number
degrees
m/s
% ground contact phase
